# Cr_2_Se_3_: a non–van der Waals platform for designing tunable 2D magnetic materials

**DOI:** 10.1038/s41598-026-49069-y

**Published:** 2026-04-24

**Authors:** Yisehak Gebredingle, Suejeong You, Heesang Kim, Nammee Kim

**Affiliations:** 1 Department of Physics, Soongsil University, Seoul, 06978 South Korea; 2 OMEG Institute, Soongsil University, Seoul, 06978 South Korea

**Keywords:** Cr_2_Se_3_, Non-van der Waals, Multifunctional Cr_2_ X _3_, Material design, Chemistry, Materials science, Physics

## Abstract

The design of magnetic materials is often enabled by rigid crystal frameworks that permit site-selective chemical control without compromising structural integrity, as exemplified by Heusler alloys. Extending this framework-driven design paradigm to low-dimensional systems remains challenging due to the limited structural complexity of many van der Waals (vdW) magnets. Here, using first-principles density functional theory, we establish rhombohedral **Cr₂X₃** (X = S, Se, Te) as a connectivity-encoded inorganic platform for designing non–van der Waals magnetic materials. Focusing on site-selectively substituted Cr₃TMSe₆ (TM = V, Mn, W), we show that the mixed face-sharing and edge-sharing Cr–Se octahedral framework remains thermodynamically accessible and dynamically, thermally, and kinetically robust upon substitution. While the Cr–Se network continues to dominate the electronic structure, transition-metal substitution acts as a controlled perturbation that systematically reshapes magnetic ground states, exchange networks, and magnetic anisotropy through distinct orbital and spin–orbit coupling mechanisms. These results demonstrate how framework topology and site chemistry cooperatively govern magnetism in non–van der Waals layered systems, establishing Cr₂Se₃ as a generalizable platform for materials-by-design beyond conventional 2D magnets.

**Supplementary Information:**

The online version contains supplementary material available at 10.1038/s41598-026-49069-y.

## Introduction

The crystallographically defined lattice of Heusler alloys has long served as a model playground for magnetic materials design, where element-specific occupation of distinct atomic sites within a rigid structural framework gives rise to a wide range of magnetic mechanisms and phenomena^1–3^. This site-selective control of magnetism—achieved without altering the underlying lattice topology—has established Heusler compounds as a canonical example of structure-enabled tunability in solid-state magnetism, creating new classes of spintronics materials^1,4,5^. Meanwhile, the discovery of intrinsic magnetism in two-dimensional (2D) materials, such as a few atomic layers of Cr_2_Ge_2_Te_6_^6^, Fe_3_GeTe_2_^7^, and monolayer CrI_3_^8,9^, and VSe_2_^10^, has opened new opportunities for low-dimensional spintronics, where reduced dimensionality and anisotropic bonding enable emergent magnetic behavior^11–13^. However, many 2D magnets derived from vdW layered compounds suffer from limited environmental stability, rapid degradation in air, and weakened interlayer coupling, posing significant challenges for practical applications^14–16^.

Rhombohedral Cr₂X₃ (X = S, Se, Te) naturally bridge these two paradigms^17–20^. They combine a rigid, topology-defined inorganic lattice—reminiscent of Heusler-type site-specific design—with a layered, non–van der Waals geometry that supports robust low-dimensional magnetism. Built from a mixed network of face-sharing and edge-sharing CrX₆ octahedra, Cr₂X₃ hosts crystallographically inequivalent metal sites and strong covalent bonding, offering a structurally robust platform in which low-dimensional magnetism can be engineered without sacrificing lattice integrity^17,19,21–23^.

Furthermore, Cr_2_ × _3_ materials are considered self-intercalated versions of CrX_2_ (TMD – transition metal dichalcogenide) where the vdW gap is filled with a Cr atom, which then provides a substantial structural and stochiometric variation for Cr and X atom, resulting in magnetic and electronic modifications^17,19,21,22,24–30^. Jianchu C. et al. recently directly observed a self-intercalation-driven conversion of 2D VSe_2_ to 3D (three-dimensional) VSe_2_ using atomic-resolution scanning tunneling electron microscopy, in which vanadium ions were observed to migrate into van der Waals gaps during the 2D-to-3D transition^27^. Besides, in Cr_2_ × _3_, the active site of these modifications is the Cr-deficient/vacant layer where stacking variant causes composition variation; 1:1 as in CrX, 1:2 as CrX_2_, 7:8 as in Cr_7_ × _8_, 3:4 as in Cr_3_ × _4_, 1:3 as in CrX_3_, 2:3 as in Cr_2_ × _3_, 5:8 as in Cr_5_ × _8_, and so on ^21,22,29,31–34^. In this sense, Cr₂X₃ functions as a connectivity-encoded inorganic playground, where magnetic exchange, electronic structure, and spin–orbit–driven anisotropy can be systematically tuned through site-selective chemical modification under strong structural constraints. This design philosophy extends the framework-driven tunability of Heusler alloys into the realm of non–van der Waals 2D magnetic materials.

In this work, we employ first-principles density functional theory to investigate site-selectively substituted Cr₃TMSe₆ (TM = V, Mn, W) compounds derived from the Cr₂Se₃ host lattice. **By combining thermodynamic, dynamical, thermal, and kinetic stability analyses with detailed electronic, magnetic, and orbital-resolved investigations, we demonstrate that the Cr–Se framework remains the dominant electronic backbone across the series, while transition-metal substitution acts as a controlled perturbation that reshapes magnetic ground states, exchange networks, and magnetic anisotropy through distinct microscopic mechanisms**. These results establish rhombohedral Cr₂Se₃ as a robust and generalizable inorganic playground for designing multifunctional non–van der Waals magnetic materials, extending framework-based magnetic design principles into the low-dimensional regime.

## Computational details

First-principles calculations were performed within density functional theory (DFT) using the Vienna ab initio Simulation Package (VASP)^35^. The projector augmented-wave (PAW) method was employed, and exchange–correlation effects were treated using the Perdew–Burke–Ernzerhof (PBE) with Hubbard *U* method using generalized gradient approximation (GGA)^36,37^. (See supplementary material Fig. S1 for results of linear response method used to determine *U*_*eff*_ values of 4.43, 5.87, and 0.81 eV for V, Mn, and W, respectively.) Spin polarization was included throughout, and spin–orbit coupling (SOC) was treated self-consistently when evaluating magnetic anisotropy and orbital-resolved properties. To ensure numerical precision, the electronic wavefunctions were expanded in a plane-wave basis set with a kinetic energy cutoff of 500 eV. Structural relaxations were carried out until the residual forces on each atom were below 0.002 eV/Å and the total energy was converged within 10⁻^7^ eV. The Brillouin zone was sampled by a Monkhorst mesh of 7 × 7 × 5 *k*-points^38^. Collinear spin orientation was used for all ground-state and electronic band calculations.

Formation energies (*E*_*f*_) and energies above the convex hull (*E*_*Hull*_) were calculated to assess thermodynamic accessibility, using bulk energies of each element and against competing binaries (See Supplementary material). Dynamical stability was assessed using the finite displacement method as implemented in the PHONOPY code^39^, employing a 2 x 2 x 1 supercell to ensure the convergence of interatomic force constants. AIMD simulations were performed in the NVT ensemble at 300 K for 10 ps with a time step of 1 fs, using a Nosé-Hoover thermostat^40^. The minimum energy pathways and migration barriers for substituted atoms were calculated using the climbing-image nudged elastic band (CI-NEB) method^41^, with a force convergence threshold of 0.02 eV/A to ensure accurate barrier determination. The nearest-neighbor and next-nearest-neighbor magnetic exchange parameters were extracted using total-energy mapping onto a classical Heisenberg model $\:\left(H=-\:\sum\:{J}_{ij}{S}_{i}^\circ\:{S}_{j}\right)$ using the four-state method, where the general formula for each *J*_*ij*_ takes the form $\:\left({J}_{ij}=-\:\frac{{E}_{1}\:+\:{E}_{2}-\:{E}_{3}\:-\:{E}_{4}}{4\cdot\:\:S\cdot\:S}\right)$, and *E*_*1−4*_ are the energies ($\:{E}_{\uparrow\:\uparrow\:},\:{E}_{\downarrow\:\downarrow\:},\:{E}_{\uparrow\:\downarrow\:},{\:E}_{\downarrow\:\uparrow\:}$) of the four states corresponding to the spins of the *i*^*th*^ and *j*^*th*^ sites (S_i_ and S_j_)^42,43^.

## Result and discussion

### Structure

**Fig. 1:**
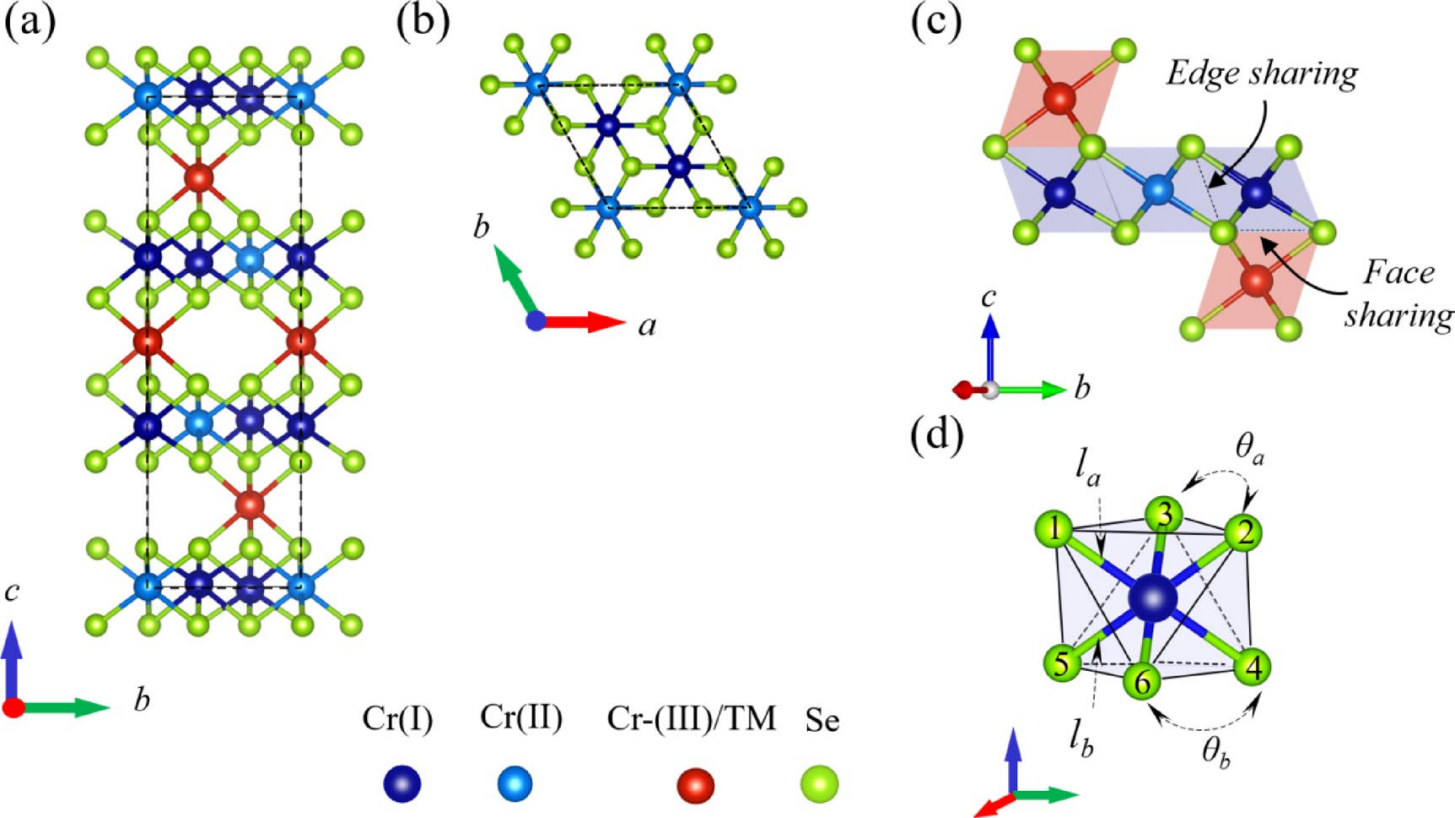
Schematic representation of the Rhombohedral Cr_3_TMSe_6_ crystal structure. (**a**) side and (**b**) top views of a unit cell. (**c**) Octahedral connectivity of face and edge sharing octahedra viewed from the reciprocal lattice a* axis, (i.e., (100)*). Two different Cr sites are indicated as light and dark blue and labeled as Cr(I) and Cr(II). (**d**) Octahedral arrangement of CrSe_6_. Se-atoms in (**c**) are numbered based on their orientation relative to the central Cr/TM-atom on the c-axis. (i.e., 1–3 above, 4–6 below Cr/TM atom) Cr–Se bond distances and Se–Cr/TM–Se bond angles are indicated by l_a_, l_b_, θ_a_, and θ_b_.

The crystallographic framework and local coordination environment of rhombohedral Cr₃TMSe₆ are shown in Fig. 1, where the layered nature and the presence of two structurally inequivalent Cr sites are highlighted. Panels (a) and (b) show the side and top views of the unit cell, where alternating fully occupied CrSe layers and TM/Cr-substituted layers form a non-van-der-Waals stacking sequence similar to the deficient-NiAs-type structures previously reported for Cr₂X₃^17,18,44–47^. The Se atoms define a hexagonal anion sublattice, while the transition-metal cations occupy octahedral voids at two distinct crystallographic positions: **Cr(I)**, located in the face-sharing octahedral columns along the *c*-axis, and Cr(II), located in the edge-sharing network within the basal plane^17^. TM atoms substitute the Cr(III) site within the face-sharing octahedral column, forming a site-selectively doped Cr₃TMSe₆ structure (Fig. 1c).

Panel (c) highlights this mixed octahedral connectivity—edge-sharing within the *a-b* plane and face-sharing between layers—an important structural motif also observed in Cr₂S₃^18^ and Cr₂Te₃^48^, where the connectivity dictates the *p-d* orbital hybridization, and therefore, the strength and sign of magnetic exchange pathways^17,23^. The arrangement of Se ligands around each Cr/TM atom is further resolved in (d), where the six coordinating Se atoms are numbered according to their position above (1–3) or below (4–6) the central octahedron along the *c*-axis. These labels correspond to the site-dependent bond distances (*l*_*a*_, *l*_*b*_) and Se–Cr/TM–Se bond angles (*θ*_*a*_, *θ*_*b*_), structural parameters that in related chromium chalcogenides are known to drive variations in crystal-field splitting, magnetic anisotropy, and the competition between direct and superexchange interactions.

**Table 1:** Structural and Thermodynamic properties; Cr_2_TMSe_3_ Ground state (GS)(Fully compensated Ferrimagnet (FCFiM) or Ferromagnetic (FM)); lattice Parameters a and c in Å; Formation Energy (E_f_) and energy above the convex hull (E_Hull_) in eV/atom for parent and substituted phases.

Crystals	GS	a	c	E_f_	E_Hull_	Stability
Cr_2_Se_3_	FCFiM	6.29	17.43	-0.531	0.00	Parent Material
Cr_3_VSe_6_	FM	6.38	17.69	-0.693	+ 0.042	Metastable
Cr_3_MnSe_6_	FM	6.31	19.03	-0.622	**+ 0.001**	**Stable**
Cr_3_WSe_6_	FM	6.38	17.32	-0.621	+ 0.108	Highly Metastable

We first examine the magnetic ground state (an energy comparison was made among four different configurations to determine the magnetic ground state as summarized in Fig. S2 and Table S1), structural and thermodynamic ground-state properties of the parent Cr₂Se₃ and the TM-substituted Cr₃TMSe₆ (TM = V, Mn, W) systems, as shown in Table 1. Upon TM substitution at the Cr(III) site, the structures retain the rhombohedral lattice symmetry of Cr₂Se₃, with only modest changes in the lattice parameters *a* and *c*. The slight lattice expansion observed for the substituted phases reflects the larger ionic radii (W-case) or distinct electronic configurations (V and Mn case) of the intercalated transition-metal ions relative to Cr. For Mn substitution, for instance, the largest expansion along the *c-axis* is observed, and the smallest expansion along the *a-b* plane is observed, while V shows a moderate increment in both lattices. On the other hand, for the W substitution case, comparably, the smallest increase is observed along the *a-b* plane while the lattice shrinks along the *c-axis*. The crystallographic Wyckoff positions of the relaxed Cr₃TMSe₆ (TM = V, Mn, W) structures are summarized in Table S2 of the Supplementary material.

For all compounds, the calculated formation energies (*E*_*f*_) are negative and comparable in magnitude to that of the base material (~ -0.6 eV), indicating thermodynamic accessibility of the substituted phases. Evaluation of the energy above the convex hull (*E*_hull_) further suggests that Cr₃MnSe₆ lies very close to the thermodynamic stability limit, while Cr₃VSe₆ and Cr₃WSe₆ are metastable and highly metastable, respectively, yet still experimentally plausible under nonequilibrium growth conditions^19,49,50^. Notably, TM substitution stabilizes a ferromagnetic (FM) ground state in all Cr₃TMSe₆ systems, in contrast to the compensated antiferromagnetic (AFM) order of pristine Cr₂Se₃ ^44,51^, demonstrating that substitution at the Cr(III) site effectively alters the balance of competing exchange interactions in chromium chalcogenides^19^.

**Table 2:** Electronic and magnetic properties; Spin-polarized band gaps (E_g(↑)_ and E_g(↓)_) in eV; and calculated magnetic moments for the Cr_3_TMSe_6_ systems. Total magnetic moment (M_tot_) (per unit cell) and Atomic Magnetic moments m_Cr(I)_, m_Cr(II)_, m_Cr(III)_, m_TM_, and m_Se_ (in Units of µ_B_) for three different Cr Sites, TM atoms, and Se chalcogenide, respectively.

Crystals	E_g(↑)_	E_g(↓)_	m_tot_	m_Cr_			m_Se_
				m_Cr(I)_	m_Cr(II)_	m_Cr(III) / TM_	
Cr_2_Se_3_	0.00	0.45	0	-2.81	2.85	2.83	0.02
Cr_3_VSe_6_	0.00	0.61	33	3.04	3.03	2.36	-0.141
Cr_3_MnSe_6_	0.00	0.97	39	2.99	3.00	4.64	-0.165
Cr_3_WSe_6_	0.08	0.45	30	2.97	3.11	1.29	-0.105

The electronic and magnetic properties of the Cr₃TMSe₆ systems are summarized in Table 2, including spin-polarized band gaps and site-resolved magnetic moments. All substituted compounds exhibit an FM ground state with sizable total magnetic moments. The calculated atomic moments indicate that Cr(I) and Cr(II) sites retain values close to 3 *µB*, consistent with high-spin Cr³⁺ in octahedral coordination. In contrast, the substituted TM site displays pronounced element-specific behavior: V carries a reduced magnetic moment consistent with its *3d²* electronic configuration, Mn exhibits an enhanced moment approaching 5 *µB* due to its half-filled *3d* shell, and W shows a strongly suppressed moment, reflecting the more delocalized and hybridized nature of its ***5d*** states, which reduces exchange splitting and spin polarization. The Se atoms acquire small induced moments of opposite sign, indicative of ***p–d*** hybridization within the face- and edge-sharing octahedral network. The spin-resolved band gaps further reveal that TM substitution tunes the electronic character from half-metallic (Cr₂Se₃/Cr₃VSe₆/Cr₃MnSe₆) to semiconducting (Cr₃WSe₆) behavior, providing a foundation for the diverse magnetic and anisotropic properties discussed in the following sections.

### Structural stability

**Fig. 2:**
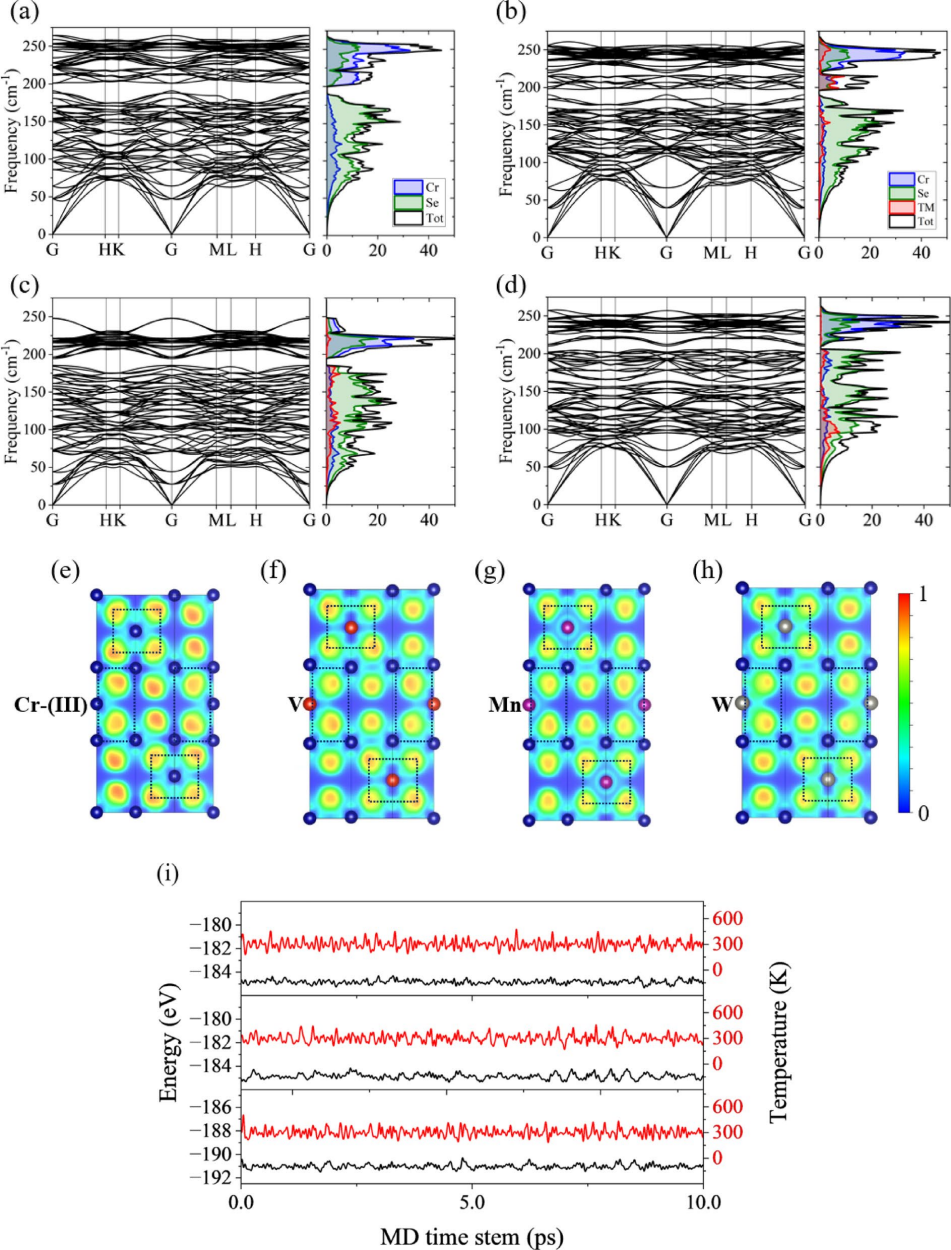
(**a**–**d**) Phonon dispersion relations and corresponding atom-projected phonon density of states for (**a**) Cr_2_VSe_3_, (**b**) Cr₃VSe₆, (**c**) Cr₃MnSe₆, and (**d**) Cr₃WSe₆, confirming the absence of imaginary frequencies, thus dynamic stability. (**e**–**h**) Electron localization function (ELF) maps along the (110) plane for (**e**) Pristine Cr_2_Se_3_, (**f**) Cr₃VSe₆, (**g**) Cr₃MnSe₆, and (**h**) Cr₃WSe₆. Red, purple, gray, and dark blue colors represent V, Mn, W, and Cr atoms, respectively. The ELF is plotted to visualize electron localization: values close to 1 indicate localized electrons, whereas ELF ≈ 0.5 corresponds to delocalized behavior, representing a homogeneous electron gas. Dashed rectangular boxes highlight modifications to the local bonding environments around the substituted TM sites. (**i**) Ab initio molecular dynamics (AIMD) simulations (Top)Cr₃VSe₆, (Middle)Cr₃MnSe₆, and (Bottom)Cr₃WSe₆ at 300 K, demonstrating the thermal stability, with negligible fluctuations in total energy and temperature and duration time (10ps).

Given that these proposed materials have ferromagnetic spin order as their ground state, we assessed their structural stability. Figure 2(a-d) presents the phonon dispersion relations and phonon density of states (PDOS) for the pristine Cr_2_Se_3_ and TM-doped compounds. The calculated phonon spectra shown in Fig. 2(b-d) show no imaginary modes across the entire Brillouin zone, confirming the dynamical stability of all three doped structures. The corresponding PDOS reveals the expected partitioning of vibrational contributions, with Se-dominated acoustic modes at low frequencies and Cr/TM-derived optical modes at higher energies, especially for V substitution.

Figures (e–h) show the ELF maps along the (110) plane for pristine Cr₂Se₃ and TM-substituted Cr₃TMSe₆ (TM = V, Mn, W). The parent Cr₂Se₃ exhibits moderate electron localization along Cr–Se bonds, reflecting mixed ionic–covalent bonding. TM substitution systematically modifies the bonding character: V substitution slightly increases localization, Mn shows a more pronounced localization (ionic-like bonding) due to its half-filled *3d* shell, while W shows a more delocalized ELF distribution arising from strongly hybridized *5d* states^52,53^. The extended W–Se bonding (i.e., metallic-like bonding) correlates with reduced local magnetic moments and enhanced-magnetic layer interaction, highlighting the role of bonding and orbital delocalization in tuning the physical properties of Cr₃TMSe₆. Overall, the distribution of electrons within the intercalated layer can be arranged in the order W > Cr > V > Mn, suggesting the possibility of tuning magnetic layer interactions across these materials.

To further examine thermal robustness, the thermal stability was assessed via AIMD simulation of Cr₃VSe₆ at 300 K for 10 ps (Fig. 2(i)). The total energy exhibits only minor fluctuations (median energy fluctuations per atom < 22 meV) around the equilibrium value, and the instantaneous temperature remains stable throughout the trajectory, indicating excellent thermal stability without structural degradation. Together, the phonon, ELF, and AIMD results demonstrate that all Cr₃TMSe₆ compounds are dynamically and thermally stable, validating their suitability for further investigations of their electronic, magnetic, and transport properties.

### NEB transition state search

**Fig. 3:**
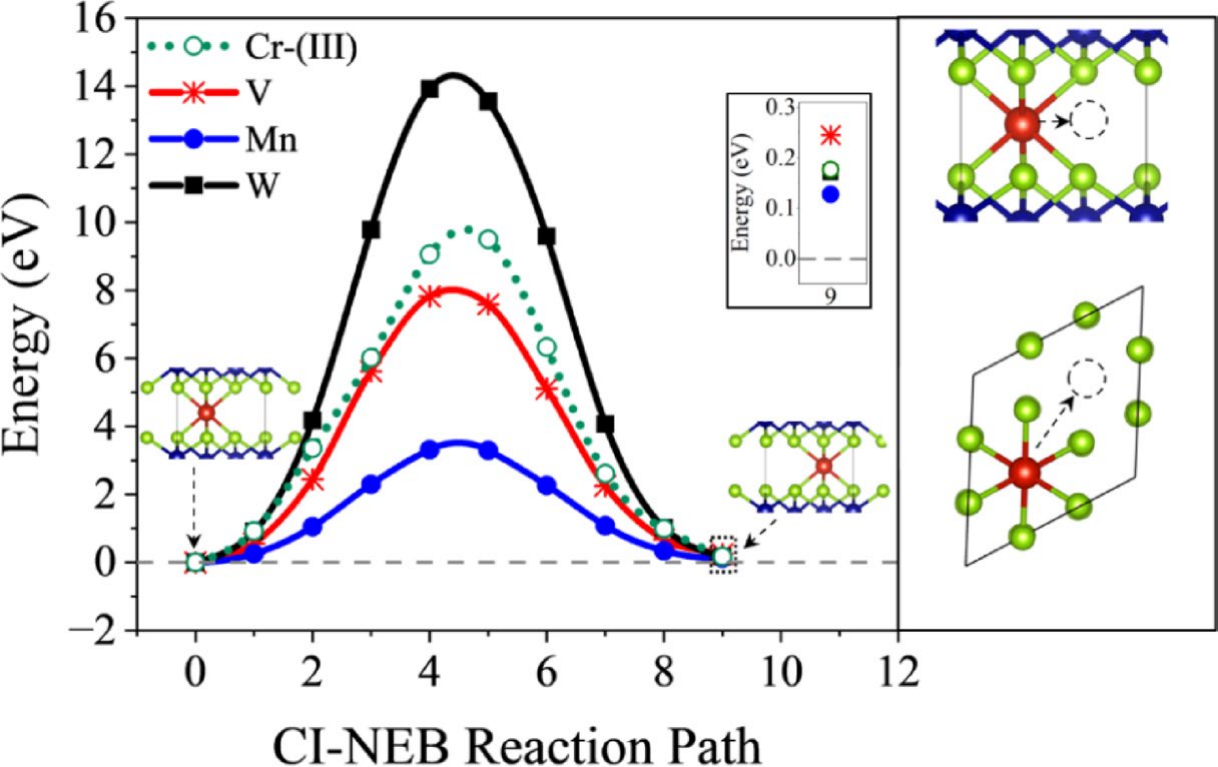
Climbing-image nudged elastic band minimum-energy pathways demonstrating the kinetic robustness of Cr₂Se₃-derived magnetic frameworks – Cr₃TMSe₆ (TM = V, Mn, W). The reaction coordinate connects the initial and final configurations illustrated by the inset structures. The dotted green line profile indicates the parent material, Cr_2_Se_3_, with Cr-(III) atoms migrating. The arrows on the left show the migration paths to the nearest vacant octahedral site.

To further validate the stability of the proposed materials against the kinetic saddle points and compare with that of the base material, we performed a transition-state search between adjacent coordination sites of the intercalated atoms. The energy barrier for this transition was calculated using the climbing-image nudged elastic band (CI-NEB) method^54,55^. Eight images were inserted, and the energy differences between the eight structures were calculated, as shown in Fig. 3.

The CI-NEB energy barriers show a clear dependence on the transition-metal constituent, with Mn exhibiting the lowest barrier and W the highest, consistent with increased lattice rigidity and stronger TM–Se bonding and the electronegativity order of W (2.36) > Cr (1.66) > V (1.63) > Mn (1.55)^56^. The uniformly large barriers (> 1 eV; significantly exceeding thermal energy at room temperature, *K*_*B*_*T*~0.026 eV) indicate that local structural rearrangements of the Cr₃TMSe₆ framework are kinetically suppressed, suggesting that these metastable configurations are experimentally accessible via nonequilibrium synthesis methods such as molecular epitaxy (MBE) or chemical vapor deposition (CVD)^19^. As shown in the inset, the octahedrally coordinated final states lie only slightly higher in energy than the initial Cr₂Se₃-derived configuration, indicating that the preservation of the lattice motif is governed **primarily by kinetic constraints**, showing why, in some cases, there have been reports of off-site placements of Cr(III) atom in the pristine material in vacant sites^57^. These results identify Cr₂Se₃ as a structurally robust archetype for stabilizing emergent magnetic materials.

### Local distortions and octahedral asymmetry

To see the effect of structural adjustments in the parent material, we have taken a close look at the local distortions around intercalated TM atoms. Table 3 provides the complete set of site-resolved octahedral parameters for each compound. In pristine Cr₂Se₃, the Cr(I) site exhibits slight asymmetry between Cr-Se bonds (*l*_*a*_*/l*_*b*_ = 2.527/2.531 Å) and between the corresponding Se–Cr–Se angles (*θ*_*a*_*/θ*_*b*_ = 92.46/89.84 Å), whereas the Cr(II) and Cr(III) sites remain nearly in a symmetric octahedra^44^.

Upon TM substitution of the intercalated layer, the Cr(I)-Se_6_ octahedral parameters evolve in a dopant-specific manner. Cr₃VSe₆ maintains relatively small elongations, with *l*_*a*_*/l*_*b*_ = 2.52/2.55 Å, and these elongations are compensated by a large angular contraction, indicating only moderate perturbation of the local ligand field. Cr₃MnSe₆, however, exhibits a more pronounced bond asymmetry and elongation (*l*_*a*_*/l*_*b*_ = 2.51/2.56 Å) and yet comparatively less reduction in angular deviation (θ_a_ = 94.39°, θ_b_ = 89.52°), suggesting a strong Jahn–Teller-like influence associated with Mn^+ 3^ *3d* shell (*t*_*2g*_^3^*e*_*g*_^1^ in octahedral coordination, resulting in asymmetric lattice distortions^58,59^. Due to the highly localized nature of *3d*-Mn states and the low electronegativity of the Mn atom, the bond length for Mn-Se is set as 2.7 Å. Consequently, this situation results in asymmetric Mn-Se (2.73 Å) and Cr-Se (2.53 Å) bond lengths, a nearly + 8% increase (from the neighboring Cr(I)/(II)-Se bond), causing significant crystal-field anisotropy around the Se atoms. This effect of MAE will be discussed in the sections followed. Meanwhile, Cr₃WSe₆ shows the smallest deviation from the parent compound, with *l*_*a*_*/l*_*b*_ = 2.55/2.54 Å and comparatively isotropic angles (θₐ = 94.68°, θ_b_ = 92.32°), reflecting the more delocalized nature of W-*5d* orbitals and their weak contribution to local anisotropy.

**Table 3:** Site-dependent octahedral parameters: Cr/TM–Se Bond Length l_a_ (Se = Se_1,2,3_) and l_b_ (Se = Se_4,5,6_) (Å) and Se–Cr/TM–Se Bond Angle θ_a_ (Se = Se_1,2,3_) and θ_b_ (Se = Se_4,5,6_) for Cr–(I), Cr–(II), and Cr–(III)/TM (V, Mn, W) sites in Cr₂Se₃ base material and the proposed Cr₃TMSe₆ crystals.

Crystals	l_a_ / l_b_			θ_a_ / θ_b_		
	Cr-(I)	Cr-(II)	Cr-(III) / TM	Cr-(I)	Cr-(II)	Cr-(III) / TM
*Cr_2_Se_3_	2.527/2.531	2.53	2.54	92.46/89.84	91.19	89.47
Cr_3_VSe_6_	2.52/2.55	2.53	2.61	96.45/89.83	93.02	87.69
Cr_3_MnSe_6_	2.51/2.56	2.51	2.73	94.39/89.52	91.69	82.94
Cr_3_WSe_6_	2.55/2.54	2.53	2.59	94.68/ 92.32	93.32	90.09

**Fig. 4:**
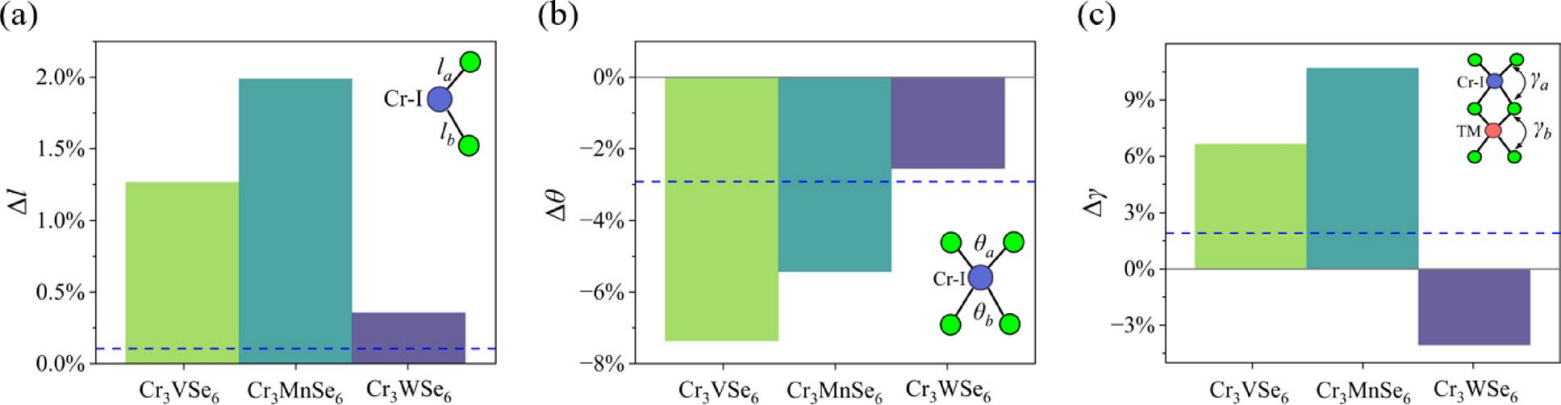
Structural distortion of the CrSe₆ octahedron around the Cr-I site in Cr₃VSe₆, Cr₃MnSe₆, and Cr₃WSe₆.(**a**) Bond-length asymmetry Δl = (l_b_/l_a_ − 1), (**b**) bond-angle asymmetry Δθ = (θ_b_/θ_a_ − 1), and (**c**) out-of-plane angular distortion Δγ = (γ_b_/γ_a_ − 1) between the two symmetry-inequivalent sets of CrSe_6_ neighbors (labeled in the insets). The dashed lines indicate the corresponding values for the base material Cr₂Se₃ (Cr-(III)–Se). Note that across all TM-intercalated systems, only the Cr-I–Se environment exhibits noticeable distortion, whereas the TM–Se and Cr-II–Se octahedra remain symmetric, as shown in the site-resolved data in Table 2.

The degree of local octahedral distortion at the Cr(I) site upon TM substitution, expressed using bond-length asymmetry Δ*l*, bond-angle asymmetry *Δθ*, and the out-of-plane angular deviation *Δγ*, is measured in Fig. 4. Here, *Δγ* quantifies the inter-octahedral tilt mismatch along the *c*-axis, capturing the relative angular misalignment between the Cr-(I)–Se₆ and TM–Se₆ octahedra that arises in the layered structure (Fig. 4(c)). In pristine Cr₂Se₃, the **Cr-(I)**-centered octahedron is only weakly distorted, as indicated by the dashed reference lines. TM substitution systematically enhances or suppresses this asymmetry.

For Cr₃VSe₆, a moderate increase in both *Δl* and *Δθ* reflects a slight imbalance between the top and bottom Se environments, while a positive *Δγ* shows a slightly “thicker” VSe_6_ intercalate layer, which hints at a possible decreased direct layer interaction. Cr₃MnSe₆ exhibits the largest local distortion among the doped compounds, with *Δl* exceeding 2% and *Δθ* approaching 6%, indicating that the high-spin Mn³⁺ configuration perturbs the Cr-S_6_ octahedral crystal field more strongly. Furthermore, a larger *Δγ* indicates a more “thicker” MnSe_6_ layer; therefore, more “separated” layers, reflecting reduced direct layer interactions (Note + 8% bond length increase mentioned above). In contrast, **Cr₃WSe₆** shows only minimal deviations from the symmetric limit, consistent with the more delocalized and uniformly distributed *5d* electronic density of W, which reduces the anisotropy in metal–ligand interactions. A negative *Δγ* indicates a thin WSe_6_ layer, possibly leading to increased layer interaction. These observations are consistent with the electron localization function (ELF) shown in Fig. 2(e-h), where the charge distribution between the layers follows the order W > V > Mn.

Importantly, the octahedral asymmetries occur only at the Cr(I) site, while the TM–Se and Cr(II)–Se octahedra remain nearly symmetric, confirming that the structural asymmetry is strongly related to the local environment of the face-sharing Cr(I) octahedron, consistent with the bonding topology (face vs. edge octahedra connectivity) shown in Fig. 1(c). These distortions provide a direct structural pathway through which the dopant/intercalant’s electronic configuration modulates the crystal-field splitting, magnetic anisotropy, and exchange interactions in Cr₃TMSe₆.

### Electronic properties: band structure and density of states

**Fig. 5:**
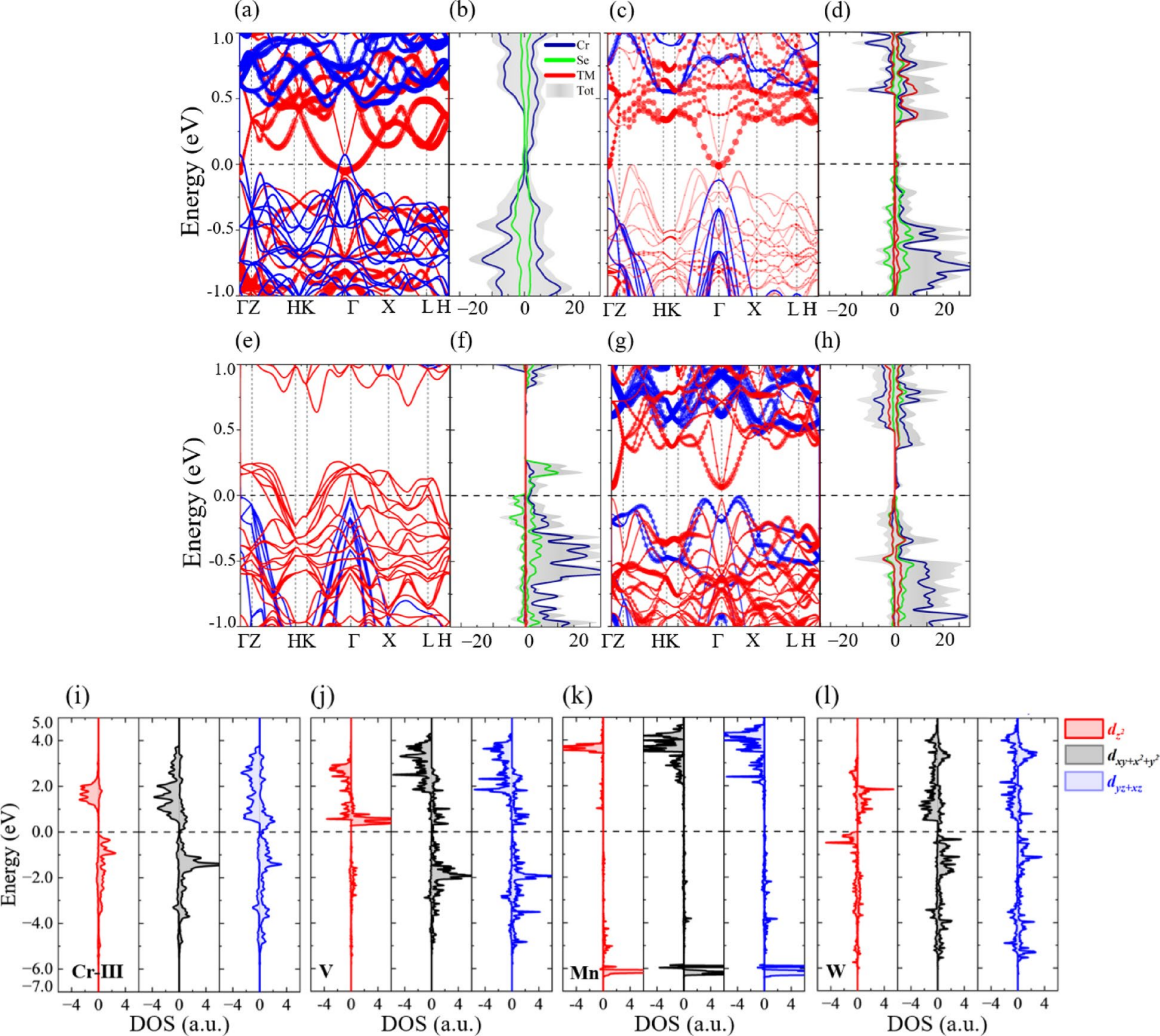
Electronic band structure of (**a**) Cr_3_VSe_6_, (**c**) Cr_3_MnSe_6_, and (**e**) Cr_3_WSe_6_ showing spin-up (red-dashed line) and spin-down (blue-solid line). Bubble-size representation qualitatively shows the strength of the 3d-3d (for Cr-V, and Mn)/ 3d-5d (Cr-W) orbital hybridization contribution from X (V, Mn, and W)-Cr atoms. The total and element-projected partial density of states of (**b**) Cr_2_Se_3_, (**d**) Cr_3_VSe_6_, (**f**) Cr_3_MnSe_6_, and (**h**) Cr_3_WSe_6_. (**i**–**l**) Orbital-resolved partial density of states (PDOS) for the dopant site in Cr₃VSe₆, Cr₃MnSe₆, and Cr₃WSe₆, respectively. The 3/5d(3p)-orbital groups—d_z_^2^​ (p_z_)(red), d_xy+x_^2^_−y_^2^ (p_y_)(gray), and d_xz+yz_ (p_x_)(blue)—are shown to highlight the orbital-selective contribution of the dopant (Se)atom near the band edges.

Next, we investigated the electronic band structures of the proposed materials. Across the Cr₃TMSe₆ series, the frontier electronic states remain dominated by the Cr–Se network (Fig. 5(a – l)), while the dopant/intercalant slightly modifies the band edges in a manner that reflects its orbital character, hybridization strength, and interaction with the layered structure (Fig. 5(i – l)). The band structures are shown in Fig. 5a, c, e, and g, with the *3d–3/5d* hybridization represented by the bubble plots.

In all pristine and TM intercalated cases, the broad distribution of *d*_*xy+x*_^*2*^_*−y*_^*2*^ *and d*_*xz+yz*_ states is in sharp contrast with a more localized *—d*_*z*_^*2*^states showing the crystal anisotropy^60^. An exception to a broad distribution of *d*_*xy+x*_^*2*^_*−y*_^*2*^ *and d*_*xz+yz*_ states is shown in Cr₃MnSe₆, where a deep valence band bond (therefore, a stronger Mn–Se bond) is formed by all highly localized *d*-states of Mn atoms and *p*-states of Se (Fig. 1(c))^61^. This explains why it is the most thermally stable candidate (Table 1) despite the longest TM-Se bond length.

Near the valence-band edge, dopant contributions follow W > V > Mn (Fig. 5 (i–l)). This arises from the greater delocalization of W 5*d* orbitals, which hybridize more strongly with the out-of-plane Se- *p*_*z*_ states (Fig. 5(l) and (Fig. S3(p)), whereas 3*d* localized orbitals of V and Mn do not. This hybridization is enhanced by the face-sharing Cr(I)Se_6_ and WS_6_ octahedra, which create a natural pathway for W–Se (*d*_*z*_^*2*^–*p*_*z*_​) coupling along the *c*-axis. Consequently, W slightly enhances the valence-band weight without dominating it. On the other hand, the extra-low states of Mn-3*d* near Fermi show the ionic bonding characters of Mn atoms, which lie far below and above Fermi.

Near the conduction-band edge, the ordering reverses to V > W > Mn, consistent with the lower energy and more localized nature of V 3*d* orbitals, and yet hybridizes effectively with the unoccupied Cr-*d* states of the upper layer (see bubble plot in Fig. 5(c)). V therefore perturbs the CBM more strongly than W, while Mn—whose 3*d* levels lie farther from both band edges—shows almost no contribution in any *d*-channel within ± 2 eV of the Fermi level, Fig. 5(k). However, a considerable enlargement of the Cr-Se bond length (Table 3; Fig. 4(a)) shifted the available states of Se-*4p* and Cr-*3d* above the Fermi level, producing a half-metallic material.

The site-resolved PDOS in Supplementary Fig. S3 further shows that Cr(I) and Cr(II) maintain similar orbital patterns across all substituents, with major differences in the Cr(I) *d*_*z*_^*2*^ ​ channel, consistent with the octahedra connectivity differences of two sites (Fig. 1(c)). The *d*_*z*_^*2*^ channel remains the major contributor to the states near the Fermi level in both V- and W-substituted cases. However, the enormously elongated bond lengths (*l*_*a/b*_) around Cr(I) in the Mn intercalant case show an orbital contribution shift to *d*_*x*_^*2*^_*−y*_^*2*^, *d*_*xz−yz*_, and *p*_*x/y*_​ channel near VBM. Meanwhile, the Se *p*-orbitals retain similar overall profiles across the three systems but show slightly enhanced *p*_*z*_​ weight near the VBM in the W case, directly reflecting stronger W–Se out-of-plane hybridization.

Together, these observations demonstrate that intercalant effects in Cr₃TMSe₆ arise from a combination of orbital-energy alignment, ***d***-state localization vs. delocalization, and hybridization anisotropy associated with the layered octahedral geometry. Despite these modifications, the Cr–Se framework remains the principal determinant of the electronic structure, and the intercalants act as **subtle**,** site-selective perturbations rather than band-edge–forming species**. This confirms the structural stability of the host material, Cr_2_Se_3_.

### Magnetic anisotropy energy (MAE)

**Fig. 6:**
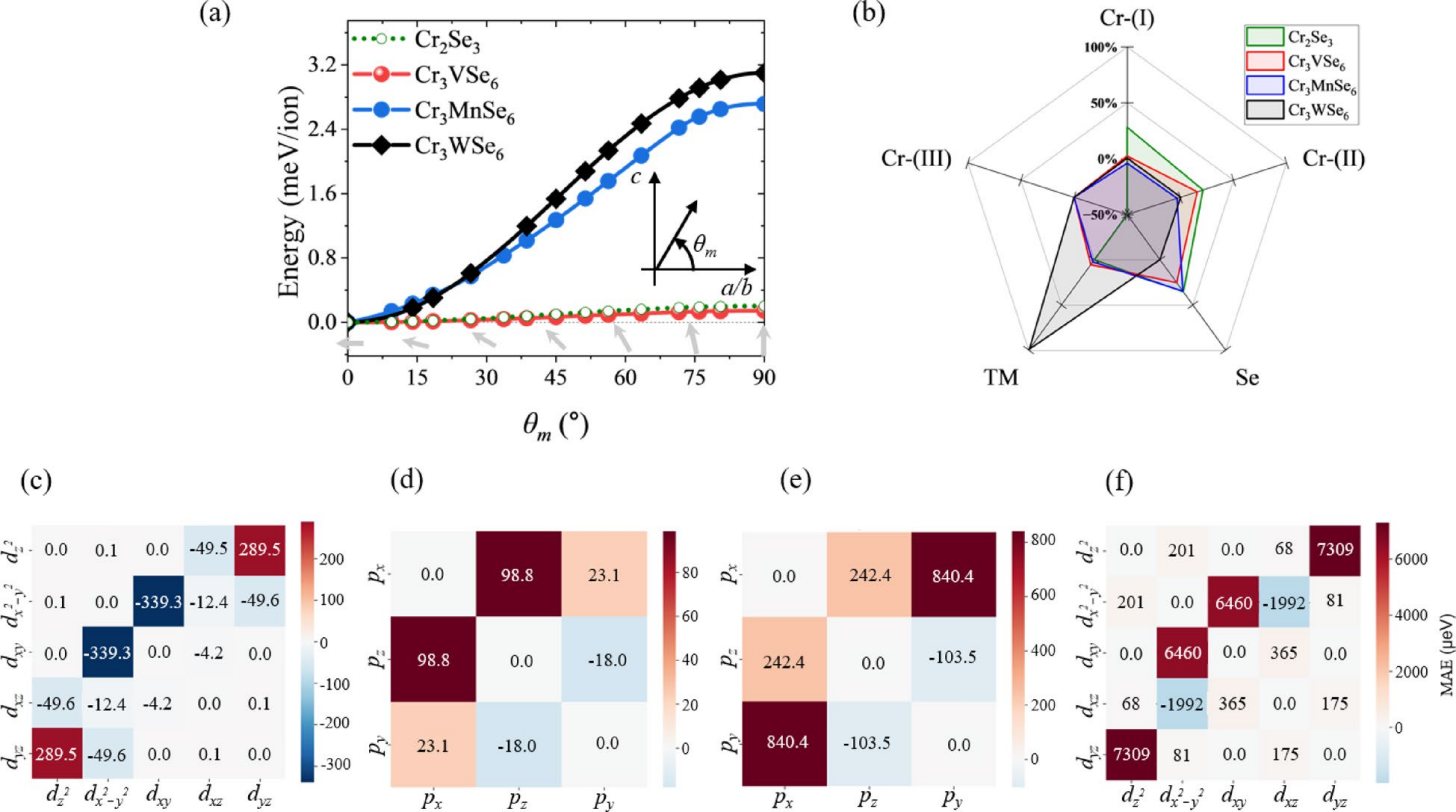
(**a**) Angular dependence of the magnetic anisotropy energy (MAE) per magnetic ion for Cr₃TMSe₆ (TM = V, Mn, and W). The inset illustrates the magnetization rotation angle (θ_m_) between the in-plane and out-of-plane directions. (**b**) The relative atomic contributions of Cr(I, II, and III), Se, and the transition metal (TM) to the total MAE. (**c**–**f**) Orbital-resolved spin–orbit coupling (SOC) energy matrices for p-orbitals in (**d**) Cr_2_Se_3_ (Cr-III), (**d**) Cr₃VSe₆ (Se-II), (**e**) Cr₃MnSe₆ (Se-I), and (**f**) W d-orbitals, highlighting the dominant SOC channels responsible for the observed anisotropy. (See Supplementary Fig. S4)

Upon inclusion of SOC and finding the easy axis for the compounds, the *in-plane* easy axis is predicted for all considered cases, preserving the pristine Cr_2_Se_3_ case, as shown in Fig. 6. Note that there have been contradicting reports of MAE in Cr_2_Se_3_ where it lies in the *a-b* plane and along the c-axis^44,57,62,63^, showing the complexity and instability of the magnetic structure varying with thickness and growth condition, and/or intrinsic strain caused by the substrate. The total MAE increases strongly from V to Mn and reaches its maximum for W substitution (Fig. 6(a)). (Note pristine Cr_2_Se_3_ has MAE of 0.2 *meV/ion*). To clarify the microscopic origin of this trend, we decomposed the total anisotropy into atom and site-specific contributions (Fig. 6(b) and Fig. S4). This analysis shows that Mn substitution yields the largest Se-derived MAE, with all three Se sites contributing equally and significantly (Fig. S4(c)). In contrast, V leads to moderate and unequal Se contributions, while W shows only small and unequal Se-site contributions, with the large total MAE of Cr₃WSe₆ arising almost entirely from the W site itself, rather than Se. For both Mn- and V-substitution, the increase in Se contribution indicates that substituent-driven changes in the Se electronic environment amplify the magnetic anisotropy.

The orbital-resolved SOC matrices in Fig. 6(c–f) clarify the electronic origin of these trends. Pristine Cr_**2**_Se_**3**_ has its major contribution from the *d*-orbital SOC of the Cr ions in the intercalated layers, i.e., Cr(III), particularly between *d*_*z*_^*2*^– *d*_*yz*_ *and d*_*xy*_ *– d*_*x*_^*2*^_*−y*_^*2*^​ channels, oppositely contributing. In the V- and Mn-substituted systems, the Se ***p***-orbital SOC matrices exhibit pronounced off-diagonal couplings, indicating that Se-mediated SOC channels play an active role in enhancing the MAE. In contrast, the W matrix displays exceptionally large, strong W *d*-orbital SOC couplings, particularly between *d*_*z*_^*2*^– *d*_*yz*_ *and d*_*xy*_ *– d*_*x*_^*2*^_*−y*_^*2*^​ channels, explaining why the MAE of Cr₃WSe₆ is dominated by the W site rather than by ligand-mediated effects.

The differing balance between ligand-mediated and TM-intrinsic SOC across the series is further influenced by local structural distortions of the octahedral framework. In Cr₃MnSe₆, pronounced bond-length asymmetry around the Se ligands (Fig. 4) enhances site-dependent hybridization, enabling comparable MAE contributions from all three Se sites. This structural activation of Se-mediated SOC is absent in Cr₃WSe₆, where the MAE remains large despite weak Se participation, owing to the dominant atomic SOC of W. Together, these results establish that Mn enhances MAE primarily through distortion-activated Se channels, whereas W achieves large anisotropy through its strong intrinsic SOC.

### Magnetic interactions

**Fig. 7:**
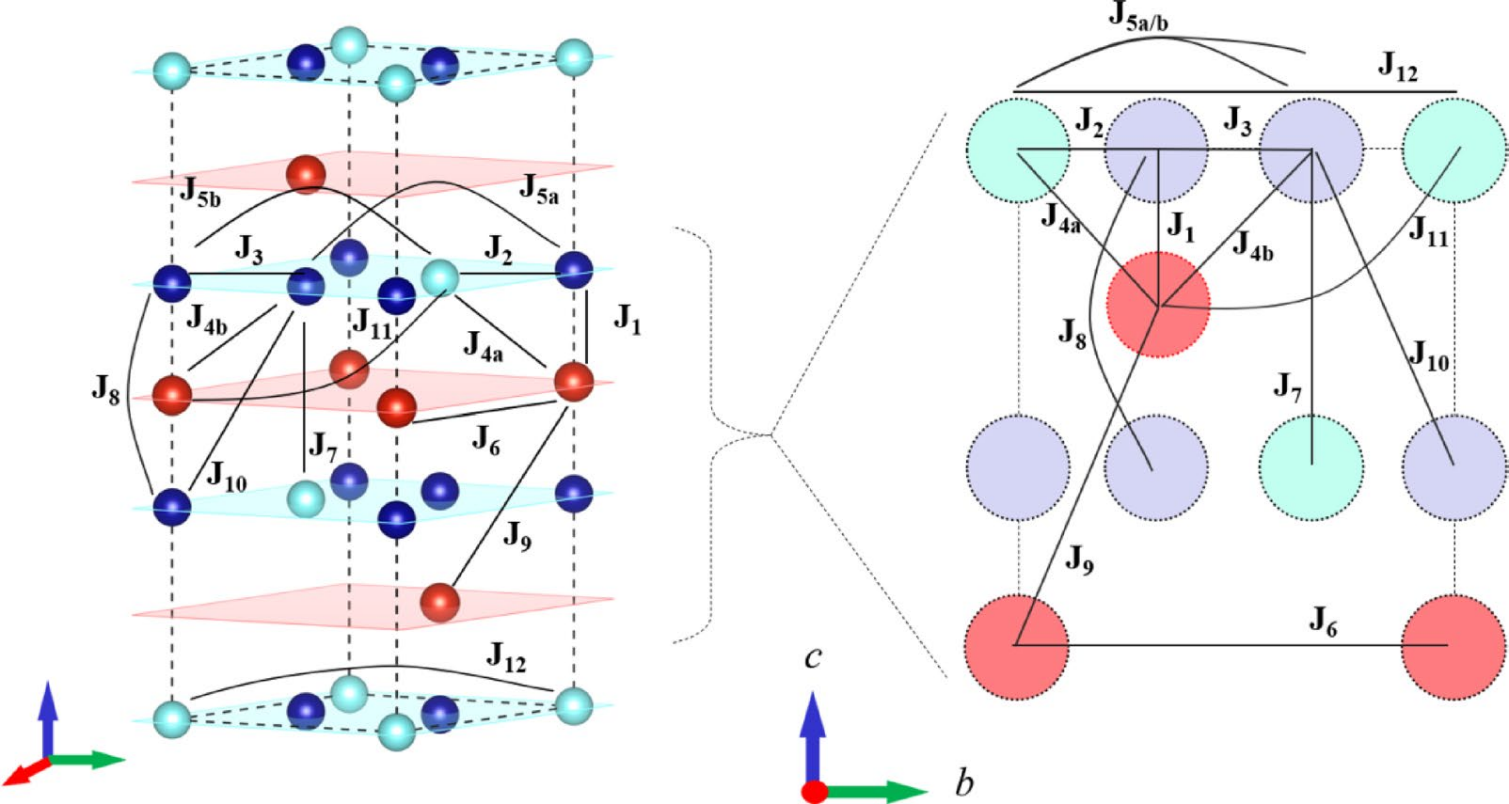
Schematic representation of main magnetic exchange pathways (J₁–J₁₂) between Cr and TM sites across intra- and inter-layer regions, (left-3D representation in a unit cell, and right-2D representation). Cr(I), Cr(II), and Cr(III)/TM magnetic ions are represented in light blue, cyan, and red, respectively.

It is known that the parent material Cr₂Se₃ exhibits a complex magnetic non-collinear structure^47,62,64,65^. To elucidate the microscopic magnetic interactions governing the ground-state magnetism of Cr₂Se₃ and Cr₃TMSe₆ (TM = V, Mn, W), we extracted the magnetic exchange parameters (*J₁–J₁₂*) using total-energy mapping. Figure 7; Table 4 show that V, Mn, and W substitutions modify the intralayer and interlayer exchange network of Cr₃TMSe₆ in distinct ways. The parent compound Cr₂Se₃ exhibits a complex exchange network characterized by several sizable antiferromagnetic interactions spanning both intra-layer and inter-layer pathways, consistent with its frustrated antiferromagnetic ground state^47,64^.

Upon TM substitution, the exchange landscape is substantially modified, with pronounced changes in both the sign and magnitude of multiple exchange channels. In many of the interlayer and intralayer interactions, the AFM-dominated interaction goes in line with a small energy difference in four different spin structures considered in determining the FM ground state (Table S1). In Cr₃VSe₆ and Cr₃MnSe₆, several competing antiferromagnetic interactions are weakened or reversed, leading to a reduced degree of magnetic frustration and stabilization of ferromagnetic ordering. By contrast, Cr₃WSe₆ displays a distinct exchange hierarchy in which a limited number of interactions dominate (*J*_*5*_ and *J*_*8*_), while many competing pathways are strongly suppressed, reflecting the influence of extended *5d* orbitals and enhanced metal–ligand hybridization along the *c-axis*. The presence of both intra-layer and inter-layer exchange couplings underscores the three-dimensional connectivity of the magnetic network despite the layered structural motif.

Overall, these results demonstrate that TM substitution fundamentally reconstructs the magnetic exchange topology of the Cr₂Se₃ host lattice, providing a microscopic foundation for the TM-dependent magnetic ground states observed in Cr₃TMSe₆ and indicating a possible tuning of the magnetic ground state. The magnetic transition from antiferromagnetic Cr₂Se₃ to ferromagnetic Cr₃TMSe₆ (TM = V, Mn, W) can be understood in terms of the competition among direct exchange, superexchange, and dopant-mediated exchange interactions. In pristine Cr₂Se₃, the magnetic ordering is dominated by antiferromagnetic Cr–Se–Cr superexchange^44^. Upon TM substitution, additional Cr–TM exchange channels emerge, and a double-exchange-like mechanism promotes ferromagnetic alignment, in which carrier-mediated hopping between Cr and TM sites favors parallel spins to maximize the kinetic energy gain^66–68^. Simultaneously, substitution modifies local bond angles and hybridization strengths, weakening the competing antiferromagnetic superexchange interactions. As a result, TM-substitution-induced hybridization enhances ferromagnetic exchange channels and suppresses antiferromagnetic coupling, stabilizing ferromagnetic ordering in Cr₃VSe₆, Cr₃MnSe₆, and Cr₃WSe₆.

**Table 4:** Summary of magnetic exchange interaction parameters (J₁–J₁₂) for Cr₂Se₃ and Cr₃TMSe₆ (TM = V, Mn, W). The interactions include both intra-layer and inter-layer exchange pathways. Positive (negative) values correspond to FM (AFM) coupling. A representative schematic of the exchange network is shown above.

Crystals	J_1_	J_2_	J_3_	J_4a_	J_4b_	J_5a_	J_5b_	J_6_	J_7_	J_8_	J_9_	J_10_	J_11_	J_12_
Cr_2_Se_3_	-4.73	0.03	-1.52	-2.50	-8.05	-4.68	-0.65	-6.18	-4.68	-0.28	-6.15	-1.99	-8.05	-2.50
Cr_3_VSe_6_	-3.53	0.82	1.15	-0.19	0.08	-3.72	-1.14	-1.78	-3.76	-0.8	-1.81	-3.28	0.12	-0.17
Cr_3_MnSe_6_	-0.60	-0.51	0.83	0.05	0.01	-1.10	-1.27	0.10	0.10	-0.16	0.06	0.1	-0.04	0.21
Cr_3_WSe_6_	-0.59	-1.53	2.04	-2.01	-1.57	4.35	0.83	0.87	0.26	-12.7	0.48	0.26	0.67	-0.09

Although Curie temperatures can, in principle, be estimated from calculated exchange parameters, the present Cr₃TMSe₆ systems exhibit multiple inequivalent exchange interactions and mixed magnetic sublattices (Cr and TM). Therefore, a quantitatively reliable prediction of $\:{T}_{C}$would require constructing the full exchange matrix and a statistical treatment, such as Monte Carlo simulations or multi-sublattice mean-field analysis. For reference, the parent compound Cr₂Se₃ exhibits antiferromagnetic ordering with a Néel temperature of approximately 50 K^47^, providing an energy scale for the magnetic interactions in this family. A rigorous evaluation of ordering temperatures for the substituted compounds is left for future work.

###  Monolayers of Cr₃TMSe₆

Knowing these materials are layered and can be synthesized down to a monolayer, as is the case with the pristine Cr_2_ × _3_, we now move to the monolayers of the proposed materials^69–73^. Table 5 summarizes the structural, electronic, and magnetic properties of bulk and monolayer (ML) Cr₂Se₃ and Cr₃TMSe₆ (TM = V, Mn, W). Upon dimensional reduction to the monolayer, all systems remain ferromagnetic (except for Cr₂Se₃*–* ferrimagnetic (see supplementary material Fig. S5)^74^ and exhibit substantial modifications of their crystal and, therefore, electronic structure, with most monolayers becoming metallic, while Cr₃WSe₆ shows a half-metallic character. This structural expansion is consistent with the reports of parent materials Cr_2_Se_3_ and Cr_2_Te_3_^45^ and strong magnetoelastic coupling predicted/observed in Cr_2_S_3_^60,75,76^.

**Table 5:** Summary table for bulk and Monolayer (ML) properties of Cr_2_TMSe_3_; Ground state (GS), lattice Parameters a and c in Å; Band type near Fermi, Band gap (Egap), and Magnetic Anisotropy Energy per unit cell (MAE) in meV/magnetic ion.

Crystals	Bulk/ML	GS	a	c	Type	E_gap_	MAE
Cr_2_Se_3_	Bulk	AFM	6.25	17.46	Half-Metallic	0.0/0.4	-0.203
	ML	FiM	6.22	17.42	Metallic	0.0/0.0	-1.85
Cr_3_VSe_6_	Bulk	FM	6.38	17.69	Half-Semi-Metallic	0.0/0.6	-0.146
	ML	FM	6.30	17.89	Metallic	0.0/0.0	-2.35
Cr_3_MnSe_6_	Bulk	FM	6.31	19.03	Half-Metallic	0.0/1.0	-2.68
	ML	FM	6.22	19.42	Metallic	0.0/0.0	-1.88
Cr_3_WSe_6_	Bulk	FM	6.41	17.24	Half-Semi-Conductor	0.08/0.5	-2.21
	ML	FM	6.38	17.52	Half-Metallic	0.0/0.2	-2.76

## Discussion summary

The physical properties of the Cr₃TMSe₆ (TM = V, Mn, W) family emerge from the interplay between the non–van der Waals layered octahedral framework of Cr₂Se₃ and TM-dependent modifications of the Cr–Se electronic backbone. The coexistence of face-sharing and edge-sharing octahedra defines directional anisotropy in *d–p* hybridization, providing a structurally encoded platform for site-selective tuning of electronic and magnetic behavior.

Electronic structure analysis shows that the frontier states remain dominated by Cr–Se orbitals across the series, confirming the robustness of the host framework. The intercalated transition metals act as orbital- and energy-selective perturbations rather than primary band-edge–forming species.

The magnetic anisotropy energy (MAE) further highlights the distinct microscopic roles of the substituent. Mn substitution activates strong ligand-mediated SOC channels through Jahn–Teller distortions associated with the high-spin Mn³⁺ (*t*_*2g*_^3^*e*_*g*_^1^ configuration, resulting in large Se-derived contributions to the MAE. In contrast, W produces the largest total MAE primarily through its strong intrinsic *5d* spin–orbit coupling and intense off-diagonal orbital mixing, despite comparatively weaker ligand participation. V, with weaker SOC and modest hybridization near the band edges, yields the smallest MAE. These results demonstrate that MAE enhancement in Cr₃TMSe₆ can arise either from distortion-activated ligand SOC or from substituent-intrinsic SOC, depending on the electronic character of the intercalant/substituent.

Magnetic exchange analysis reveals that TM substitution substantially reconstructs the exchange topology of the Cr₂Se₃ host lattice. Changes in both the sign and magnitude of multiple intra-layer and inter-layer exchange pathways reflect the sensitivity of superexchange interactions to substituent-induced modifications of orbital overlap and bonding geometry. Despite the layered structural motif, the exchange network shows substantial three-dimensional connectivity (strong inter- and intralayer interactions), underscoring the non–van der Waals nature of the system and its departure from conventional weakly coupled 2D magnets. Notably, the relatively small energy separations between competing magnetic configurations (Supplementary Table S1) indicate that the substituted systems reside near magnetic phase boundaries, suggesting that strain, dimensional confinement, or electrostatic gating may further tune the magnetic ground state^44,60^.

Finally, extension to the monolayer limit demonstrates that dimensional extension/reduction provides an additional degree of freedom for tuning electronic and magnetic properties. While the Cr–Se framework remains intact, the monolayers exhibit notable changes in band character and magnetic anisotropy, reinforcing the versatility of Cr₂Se₃ as a host platform for engineering multifunctional low-dimensional materials.

From a spintronics perspective, the coexistence of structurally tunable ligand-mediated anisotropy and intrinsic substituent-driven spin–orbit coupling provides distinct functional pathways within a single material platform. Distortion-sensitive anisotropy offers opportunities for strain- or electric-field-controlled magnetic architectures, enabling voltage-tunable magnetic architectures. In contrast, intrinsic heavy-element spin–orbit coupling provides a more robust route toward high-stability magnetic memory applications. The ability to trade tunability for robustness within the same non–van der Waals framework underscores the device versatility of Cr₂X₃-based systems for spin–orbit-engineered technologies.

**Fig. 8:**
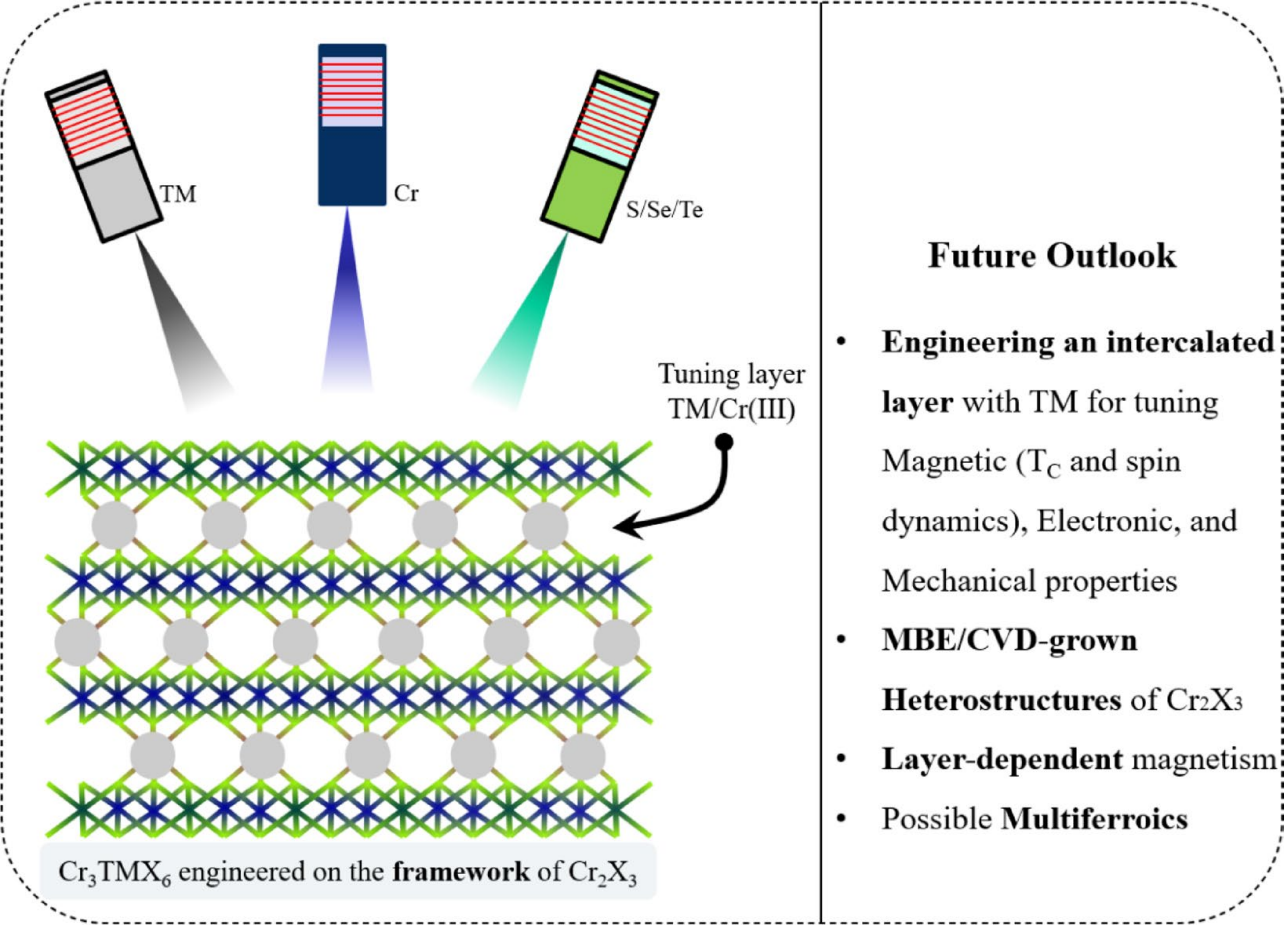
The Cr_2_X_3_ Framework: From Fundamental Design to Experimental Outlook. (Left) The schematics of Cr₂Se₃ as Structural Backbone: Cr₂Se₃ serves as a connectivity-encoded framework, with the Cr(III) layer acting as a chemically active substitution site for site-selective engineering via MBE under a layer-by-layer growth mechanism, controlled by X-to-Cr-to-TM flux conditions. (Right) Future Outlook: The structural integrity and kinetic robustness of this non-VdW system suggest high compatibility with MBE/CVD growth for large-scale synthesis. Future efforts may leverage the tunable anisotropy mechanisms identified here and the reconstructed exchange topology to engineer Curie temperatures and spin-dynamic properties in non–van der Waals heterostructures.

Overall, this work establishes non–van der Waals Cr₂Se₃—and by extension related Cr₂X₃ compounds as a robust and flexible playground for designing multifunctional magnetic materials (see Fig. 8 below). By combining site-selective TM substitution with intrinsic layered octahedral connectivity, a wide range of electronic structures, magnetic anisotropies, and exchange interactions can be realized within a single structural archetype, offering promising opportunities for future exploration of low-dimensional magnetism and spin–orbit–driven phenomena.

## Supplementary Information

Below is the link to the electronic supplementary material.

Supplementary Material 1

## Data Availability

The datasets used and/or analyzed during the current study are available from the corresponding author on reasonable request.

## Declarations

### Data-availability

The datasets used and/or analyzed during the current study are available from the corresponding author on reasonable request.

**Correspondence** and requests for materials should be addressed to Y.G. and N.K.

### Reprints and permissions information

are available at.
