# Supplementary material for: Cr2Se3: a non–van der Waals platform for designing tunable 2D magnetic materials
Source: Sci Rep. 2026 Apr 24;16:19079. doi: 10.1038/s41598-026-49069-y (PMC13279928; doi:10.1038/s41598-026-49069-y)
Supplement: Supplementary file 1 — Supplementary Material 1 [file 41598_2026_49069_MOESM1_ESM.docx]

Supplementary material

Cr₂Se₃: A Non–van der Waals platform for designing tunable 2D magnetic materials

Yisehak Gebredingle^1,2, *^, Suejeong You^1,2^, Heesang Kim^1,2^, Nammee Kim^1, *^

^1^Department of Physics, Soongsil University, Seoul 06978, South Korea
^2^OMEG Institute, Soongsil University, Seoul 06978, South Korea

*Corresponding author. Email: [yisehakgd@soongsil.ac.kr](mailto:yisehakgd@soongsil.ac.kr%20)  (Y.G.) [nammee@ssu.ac.kr](mailto:nammee@ssu.ac.kr) (N.K.)

KEYWORDS: Cr2Se_3_, Doping, non-van der Waals, Multifunctional Cr_2_X_3_, Material design

# *U_eff_* – Value Derivation: The linear response plots

**
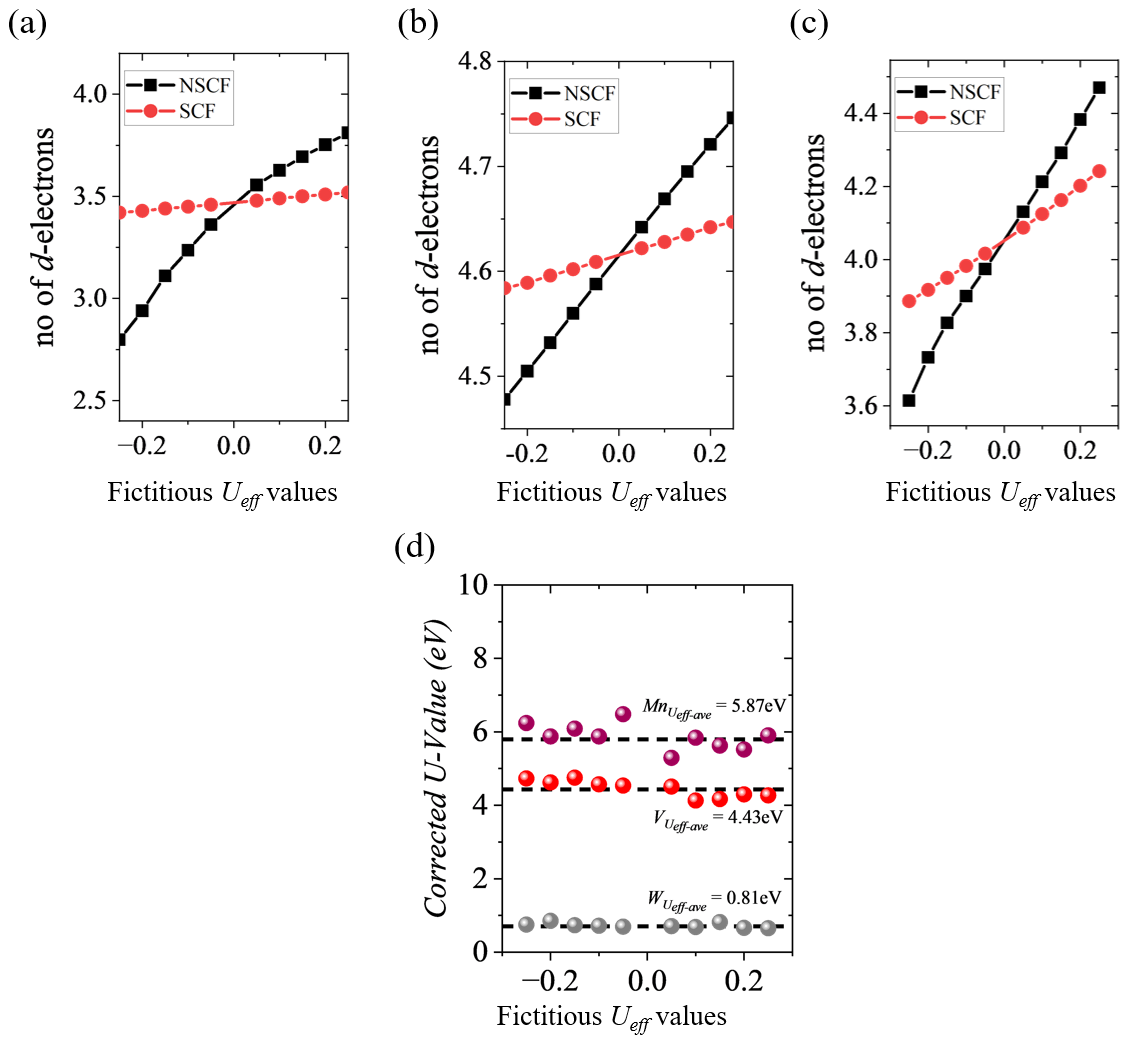
**

***Figure S1.*** *Linear response calculation of the Hubbard U parameter for substituted TM sites. Panels* ***(a–c)*** *show the variation of d-orbital occupation (Δn) with respect to the applied local potential perturbation (α) for* ***(a)*** *V,* ***(b)*** *Mn, and* ***(c)*** *W. The slopes represent the linear regression of the interacting (χ) and non-interacting (χ_0_) response functions. The effective Hubbard (U_eff_) value is calculated from the difference in the inverse slopes: U = χ^-1^ - χ_0_^-1^.* ***(d)*** *Summary of the site-specific U values used for the Cr, V, Mn, and W d-orbitals throughout the DFT+U calculations to ensure an accurate description of the localized electronic states and magnetic moments.*

# Possible spin-order for the three identified magnetic sites of Cr and TM

**
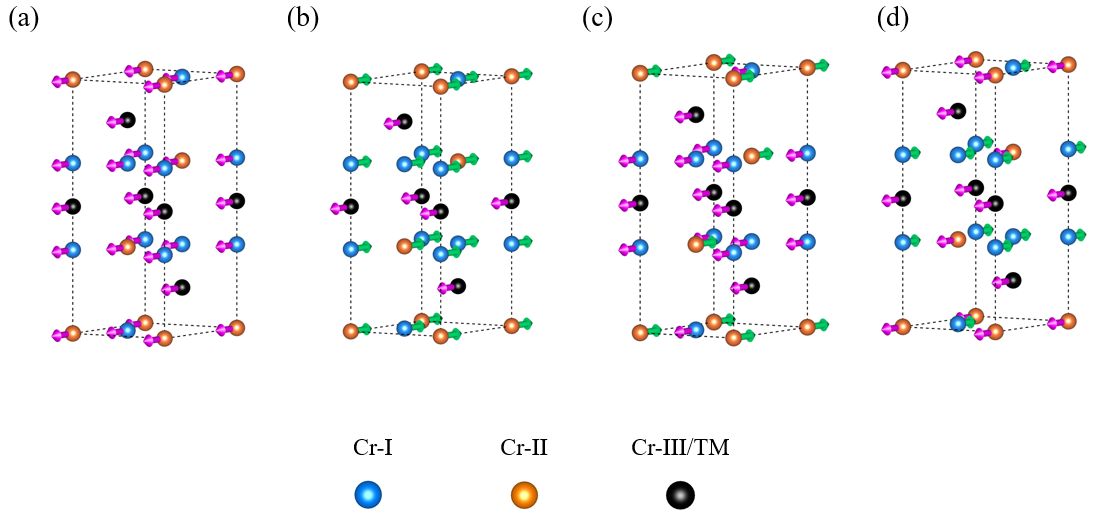
**

***Figure S2.*** *Four unique possible spin orderings of Cr and TM atoms in a rhombohedral unit cell of Cr_2_Se_3_ /Cr_3_TMSe_6_.* ***(a)*** *Ferromagnetic (↑↑↑),* ***(b)****Ferrimagnetic-II (↑↑↓),* ***(c)*** *Ferrimagnetic-I (↑↓↑), and* ***(d)*** *E-type Antiferromagnetic spin orderings. Each Cr is represented with a blue (Cr-I), orange (Cr-II), and black (Cr-III/TM) color. The green/purple arrow indicates the spin-up/down directions.*

# Ground state calculation results

***TableS1.*** *Calculated Lattice Parameters (a and c in Å); Relative energies ΔE (meV per Formula Unit, referenced to the lowest-energy magnetic configuration); Total Magnetic Moment M and Atomic Magnetic Moment m_Cr(I)_, m_Cr(II)_, m_Cr(III)_, m_Cr(TM)_, and m_Se_ (in Units of μB) for different Cr magnetic configurations of magnetic ions in Cr_2_Se_3_ and Cr_3_TMSe_6_. Note that three unique sites of magnetic ions are represented with arrow directions as shown in* ***Fig S2****.*

| **Crystals** | **Magnetic**  **ordering** | ***a*** | ***c*** | ***∆E*** | ***m_tot_*** | ***m_Cr(I)_*** | ***m_Cr(II)_*** | ***m_Cr(III)/TM_*** | ***m_Se_*** |
| --- | --- | --- | --- | --- | --- | --- | --- | --- | --- |
| Cr_2_Se_3_ (*U_eff_* = 0 eV) | FM (↑↑↑) | 6.34 | 17.67 | 9.75 | 36 | 3.08 | 3.06 | 3.15 | -0.14 |
|  | FiM-I (↑↓↑) | 6.24 | 17.87 | 28.11 | 18 | 2.95 | -2.77 | 3.07 | -0.07 |
|  | FiM-II (↑↑↓) | 6.37 | 16.91 | 19.21 | 18 | 2.91 | 2.96 | -2.78 | -0.06 |
|  | **FCFiM (↓↑↑)** | **6.25** | **17.46** | **0** | **0** | **-2.81** | **2.88** | **2.88** | **0.01** |
| Cr_3_VSe_6_ (*U_eff_* = 4.43 eV) | **FM (↑↑↑)** | **6.38** | **17.69** | **0** | **33** | **3.04** | **3.03** | **2.36** | **-0.141** |
|  | FiM-I (↑↓↑) | 6.29 | 17.90 | 164 | 15 | 2.91 | -2.82 | 2.27 | -0.07 |
|  | FiM-II (↑↑↓) | 6.36 | 17.59 | 263 | 15.87 | 2.87 | 2.88 | -2.67 | -0.06 |
|  | FiM-III (↓↑↑) | 6.28 | 17.92 | 502 | -2.79 | -2.87 | 2.79 | 2.34 | -0.04 |
| Cr_3_MnSe_6_ (*U_eff_* = 5.87 eV) | **FM (↑↑↑)** | **6.31** | **19.03** | **0** | **39** | **2.99** | **3.00** | **4.64** | **-0.165** |
|  | FiM-I (↑↓↑) | 6.16 | 19.71 | 84.08 | 22.33 | 2.85 | -2.78 | 4.65 | -0.06 |
|  | FiM-II (↑↑↓) | 6.29 | 18.81 | 119.53 | 10.31 | 2.92 | 2.86 | -4.63 | -0.06 |
|  | FiM-III (↓↑↑) | 6.16 | 19.63 | 155.22 | 7.67 | -2.78 | 2.89 | 4.64 | -0.06 |
| Cr_3_WSe_6_ (*U_eff_* = 0.8 eV) | **FM (↑↑↑)** | **6.41** | **17.24** | **0** | **30** | **3.08** | **3.08** | **1.97** | **-0.12** |
|  | FiM-I (↑↓↑) | 6.33 | 17.41 | 447.62 | 12 | 2.95 | -2.74 | 1.89 | -0.05 |
|  | FiM-II (↑↑↓) | 6.43 | 17.01 | 21.6 | 18 | 2.81 | 2.88 | -1.89 | -0.07 |
|  | FiM-III (↓↑↑) | 6.33 | 17.31 | 1537.7 | -7.28 | -2.79 | 2.88 | 1.89 | -0.02 |

# Wyckoff positions of relaxed Cr₃TMSe₆

***Table S2.*** *Wyckoff positions of relaxed Cr₃TMSe₆ (TM = V, Mn, W) structures obtained from symmetry analysis. All structures are described by the rhombohedral* ***R-3*** *space group (No. 148) listed in the hexagonal setting. Lattice parameters and representative fractional coordinates are listed below. The hexagonal angles are α = 90 °, β = 90 °, and γ = 120 °.*

| ***Compound*** | ***Lattice***  ***Parameters (Å)*** | ***Atom*** | ***Wyckoff site*** | ***Fractional coordinates*** |
| --- | --- | --- | --- | --- |
| *Cr₃VSe₆* | *a=6.385* | *Cr* | *6c* | *(0, 0, 0.32776)* |
|  |  | *Cr* | *3a* | *(0, 0, 0)* |
|  | *c=17.694* | *V* | *3b* | *(0.33333, 0.66667, 0.16667)* |
|  |  | *Se* | *18f* | *(0.00370, 0.34269, 0.25514)* |
| *Cr₃MnSe₆* | *a=6.308* | *Cr* | *6c* | *(0, 0, 0.32919)* |
|  |  | *Cr* | *3a* | *(0, 0, 0)* |
|  | *c=19.033* | *Mn* | *3b* | *(0.33333, 0.66667, 0.16667)* |
|  |  | *Se* | *18f* | *(0.00073, 0.33804, 0.25901)* |
| *Cr₃WSe₆* | *a=6.419* | *Cr* | *6c* | *(0, 0, 0.33137)* |
|  |  | *Cr* | *3a* | *(0, 0, 0)* |
|  | *c=17.110* | *W* | *3b* | *(0.33333, 0.66667, 0.16667)* |
|  |  | *Se* | *18f* | *(0.00270, 0.33986, 0.25228)* |

# Thermodynamic Stability Calculation Methodology

Formation Energy (Δ*E_f_*)

The formation energy determines the chemical stability of a compound relative to its constituent pure elements. For the Cr₃MSe₆ system (where M = V, Mn, W), the formation energy per atom is calculated as:

Δ*E_f_* = *E_atom_ (Cr₃TMSe₆)* − [0.3 *E_atom_ (Cr_bulk_)* + 0.1 *E_atom_ (M_bulk_)* + 0.6 *E_atom_ (Se_bulk_)*]

Where:

- *E_atom_(Discovery)*: Total energy from the VASP OUTCAR divided by the number of atoms.
- *E_atom_(Reference)*: Energy per atom of the most stable bulk elemental phase (i.e., Trigonal Se, BCC Cr, Mn, and W).
- **Coefficients** are based on atomic fractions (30% Cr, 10% M, 60% Se).

Energy Above the Convex Hull (*E_hull_*)

The energy above the hull (Δ*E_Hull_*) determines whether a ternary phase is stable against decomposition into competing binaries. The primary decomposition pathway is:

Cr₃TMSe₆ → 1.5 Cr₂Se₃ + TMSe₂

For V and W cases (TMSe₂ reference):

*E_hull_* = *E_atom_* (*Cr₃TMSe₆*) − [0.75 *E_atom_ (Cr₂Se₃)* + 0.3 *E_atom_* *(TMSe₂)*]

For the Mn case (MnSe reference):

*E_hull_* = *E_atom_ (Cr₃MnSe₆)* − [0.75 *E_atom_ (Cr₂Se₃)* + 0.2 *E_atom_ (MnSe)* + 0.05 *E_atom_ (Se_bulk_)*]

- Stability Criteria^1,2^
- *E_hull_* = 0: Thermodynamic ground state (stable).
- 0 < E_hull_ < 0.100 eV/atom: Metastable and likely synthesizable.
- E_hull_ > 0.100 eV/atom: Highly metastable relative to binary phases.

**Reference Values Used in This Study (DFT results)**

| Phase | Structure | Space Group | *E_tot_* (eV/atom) |
| --- | --- | --- | --- |
| Cr₂Se₃ | Rhombohedral | *R-3(148)* | -6.42748 |
| VSe₂ | 1T-TMD | *P̅3m1(163)* | -4.73333 |
| MnSe | NiAs-type | *P6₃/mmc(194)* | -5.85192 |
| WSe₂ | 2H-TMD | *P6₃/mmc(194)* | -6.41706 |
| Se_bulk_ | Trigonal | *P3₁21(152)* | -3.49822 |

Below is the breakdown of how the energy above the convex hull (*E_hull_*) was derived for the discovery phases using the reference values obtained from first-principles calculations.

## **Vanadium substitution (Cr₃VSe₆)**

- Stoichiometry:
  1.0 unit of Cr₃VSe₆ (10 atoms) = 1.5 units of Cr₂Se₃ (5 atoms each) + 1.0 unit of VSe₂ (3 atoms) + adjusted Se balance.
- Normalized Equation:

*E_hull_* = *E_atom_ (Cr₃VSe₆)* − [0.75 × *E_atom_ (Cr₂Se₃)* + 0.3 × *E_atom_ (VSe₂)*]

= −6.1981 − [0.75 × (−6.42748) + 0.3 × (−4.73333)]

= −6.1981 − [−4.8206 − 1.4200]

*E_hull_* = +0.0425 eV/atom

## **Manganese substitution (Cr₃MnSe₆)**

- Stoichiometry:
   Since the stable reference for Mn is MnSe (1:1), *Se_bulk_* is added to balance the 60% selenium requirement.
- Normalized Equation:

*E_hull_* = *E_atom_ (Cr₃MnSe₆)* − [0.75 × *E_atom_ (Cr₂Se₃)* + 0.2 × *E_atom_ (MnSe)* + 0.05 × *E_atom_ (Se_bulk_)*]

= −6.1656 − [0.75 × (−6.42748) + 0.2 × (−5.85192) + 0.05 × (−3.49822)]

= −6.1656 − [−4.8206 − 1.1704 − 0.1749]

*E_hull_* = +0.0003 eV/atom (approximated as 0.001 in Table 1 in the manuscript)

## **Tungsten substitution (Cr₃WSe₆)**

- Stoichiometry:
  1.5 units of Cr₂Se₃ + 1.0 unit of WSe₂.
- Normalized Equation:

*E_hull_* = *E_atom_ (Cr₃WSe₆)* − [0.75 × *E_atom_ (Cr₂Se₃)* + 0.3 × *E_atom_ (WSe₂)*]

= −6.6375 − [0.75 × (−6.42748) + 0.3 × (−6.41706)]

= −6.6375 − [−4.8206 − 1.9251]

*E_hull_* = +0.1078 eV/atom

# Site- and orbital-resolved projected density of states (PDOS)


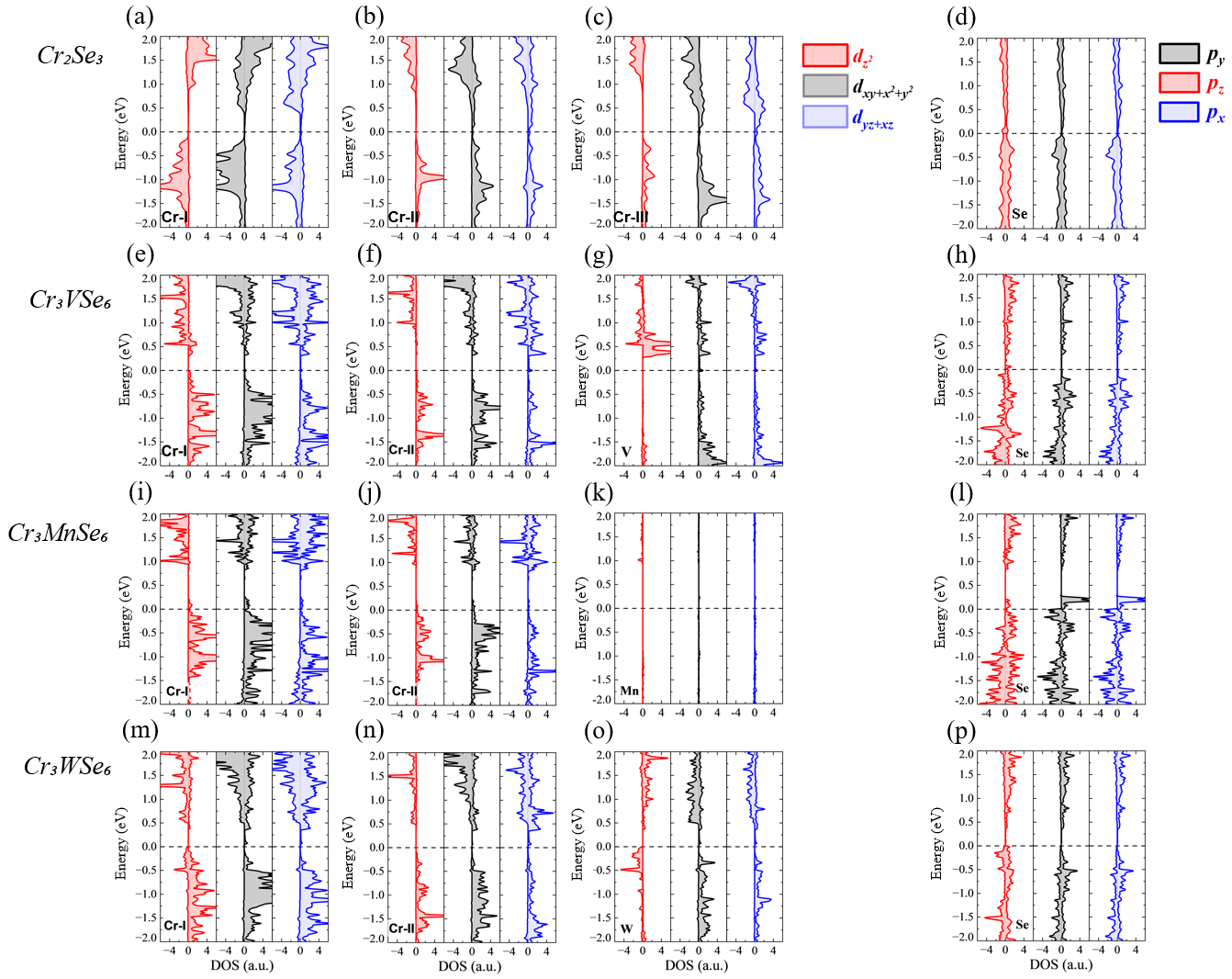


***Figure S3.*** *Site- and orbital-resolved projected density of states (PDOS) for Cr₂Se₃ and Cr₃TMSe₆ (TM = V, Mn, W). Panels* ***(a–d)*** *correspond to Cr₂Se₃;* ***(e–h)*** *Cr₃VSe₆;* ***(i–l)*** *Cr₃MnSe₆; and* ***(m–p)*** *Cr₃WSe₆. For each compound, the PDOS of inequivalent Cr sites (Cr-I, Cr-II, and Cr-III/TM), the dopant site (V, Mn, or W), and representative Se atoms are shown. The d-orbital groups—d₍z²₎ (red), d₍x²−y²₎ + d₍xy₎ (gray), and d₍xz₎ + d₍yz₎ (blue)—are plotted to highlight orbital-selective contributions near the Fermi level (set to 0 eV). The comparison illustrates substitution-dependent modifications of orbital character while confirming that the Cr–Se framework remains the dominant contributor to the frontier electronic states.*

# Atomic-site-dependent MAE


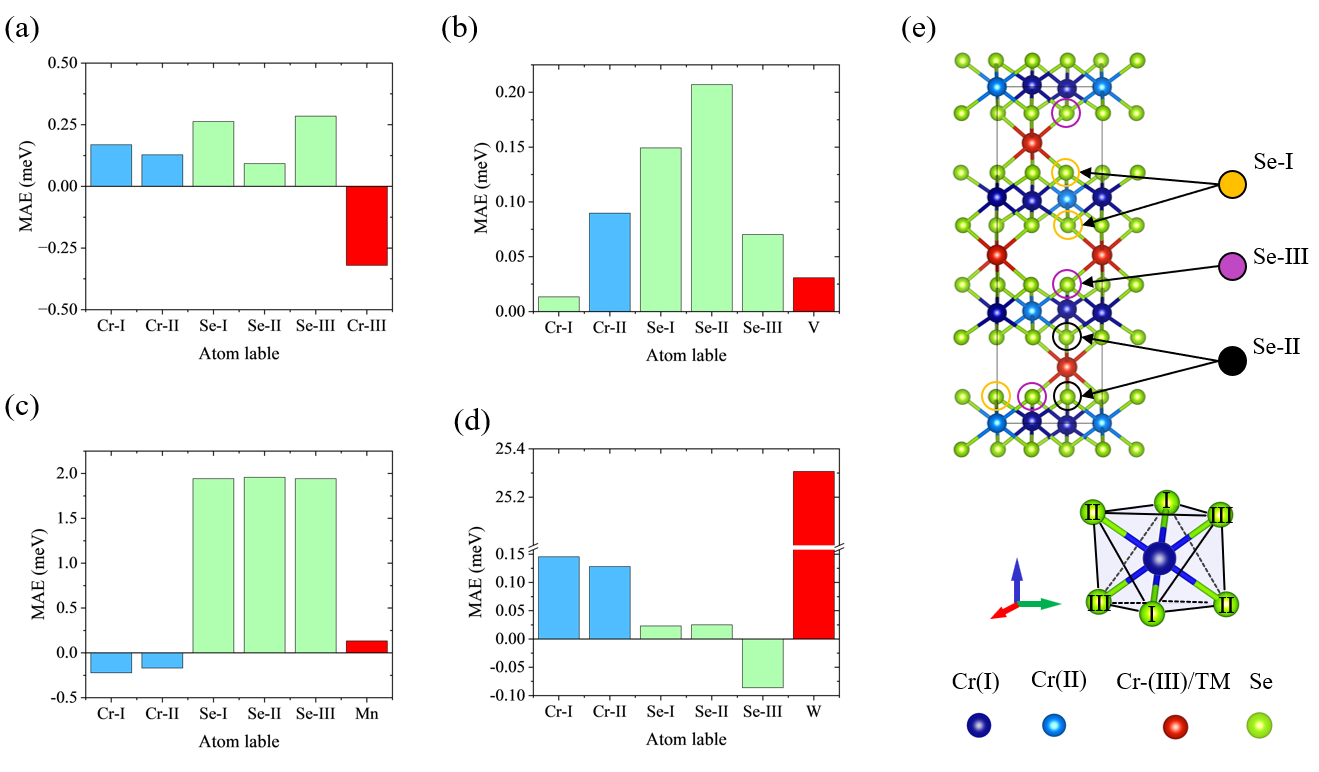


***Figure S4.*** *Atom-resolved magnetic anisotropy energy (MAE) contributions for* ***(a)*** *Cr₃VSe₆,* ***(b)*** *Cr₃MnSe₆, and* ***(c)*** *Cr₃WSe₆. Blue, green, and red bars represent the MAE contributions from Cr, Se, and TM (V, Mn, W) atoms, respectively. The results reveal a progressive enhancement of MAE from V to W substitution, driven by strong spin–orbit coupling on the heavy W site and its hybridization with neighboring Se p-orbitals.* ***(d)*** *Se atoms. The arrangement of CrSe6 is shown with opposite sides of the octahedral structure. (e) Side view of Cr_3_TMSe_6_ unit cell with Se-(I), (II), and (III) shown.*

# Details of the Four-State exchange parameters calculation method used

We map the DFT total energies of $2 \times2\times1$ supercell onto a Heisenberg Hamiltonian,

$$\left( H=- \sum J_{ij}S_{i}^{\circ}S_{j} \right)$$

Where $J_{ij}$ is the exchange interaction between magnetic sites $i$ and $j$, $S_{i}$ is the spin vector on site. To extract a specific exchange parameter $J_{ij}$ of magnetic sites $i$ and $j$, all other spins are kept fixed, and four collinear spin configurations are constructed for only these two sites:

E_↑↑​_, E_↑↓_, E_↓↑_, and E_↓↓_.

The total energies of these four states can be written as

$$E_{\uparrow\uparrow}=E_{0}-J_{ij} S_{i} S_{j}+K$$

$$E_{\uparrow\downarrow}=E_{0}+J_{ij} S_{i} S_{j}+L$$

$$E_{\downarrow\uparrow}=E_{0}+J_{ij} S_{i} S_{j}+M$$

$$E_{\downarrow\downarrow}=E_{0}-J_{ij} S_{i} S_{j}+N$$

​where $E_{0}$ collects pair-independent terms, and the extra terms K, L, M, and N arise from the interactions of sites $i$ and $j$ with the frozen surrounding spins. By combining the four energies, those background contributions cancel, giving

$$J_{ij}= \frac{E_{\uparrow\uparrow}+ E_{\downarrow\downarrow}- E_{\uparrow\downarrow}-E_{\downarrow\uparrow}}{4S_{i}\cdot S_{j}}$$

Repeating this procedure for each inequivalent magnetic pair yielded the set of exchange parameters 𝐽_1_ – 𝐽_12_.

Below is an example of *J_1_* for parent material Cr_2_Se_3_.

Total energies:

✔ $E_{\uparrow\uparrow}$ _(FM + +)_: -769.38755 eV

✔ $E_{\downarrow\downarrow}$ _(FM – –)_: -769.51206 eV

✔ $E_{\uparrow\downarrow}$ _(AFM + –)_: -769.58497 eV

✔ $E_{\downarrow\uparrow}$ _(AFM – +)_: -769.48510 eV

Calculated exchange coupling: (Spins for Cr atoms: *S_1_* = 3.0, *S_2_* = 3.0 → *S_1_·S_2_* = 9.0)

ΔE=(−0.76938755) + (−0.76951206) − (−0.76958497) − (−0.76948510) = 0.17046 eV

*J_1_* = ​$-\frac{0.17046}{4 *3*3}$ eV

*J_1_* = **−** 4.73 meV

# Monolayers of Cr₃TMSe₆


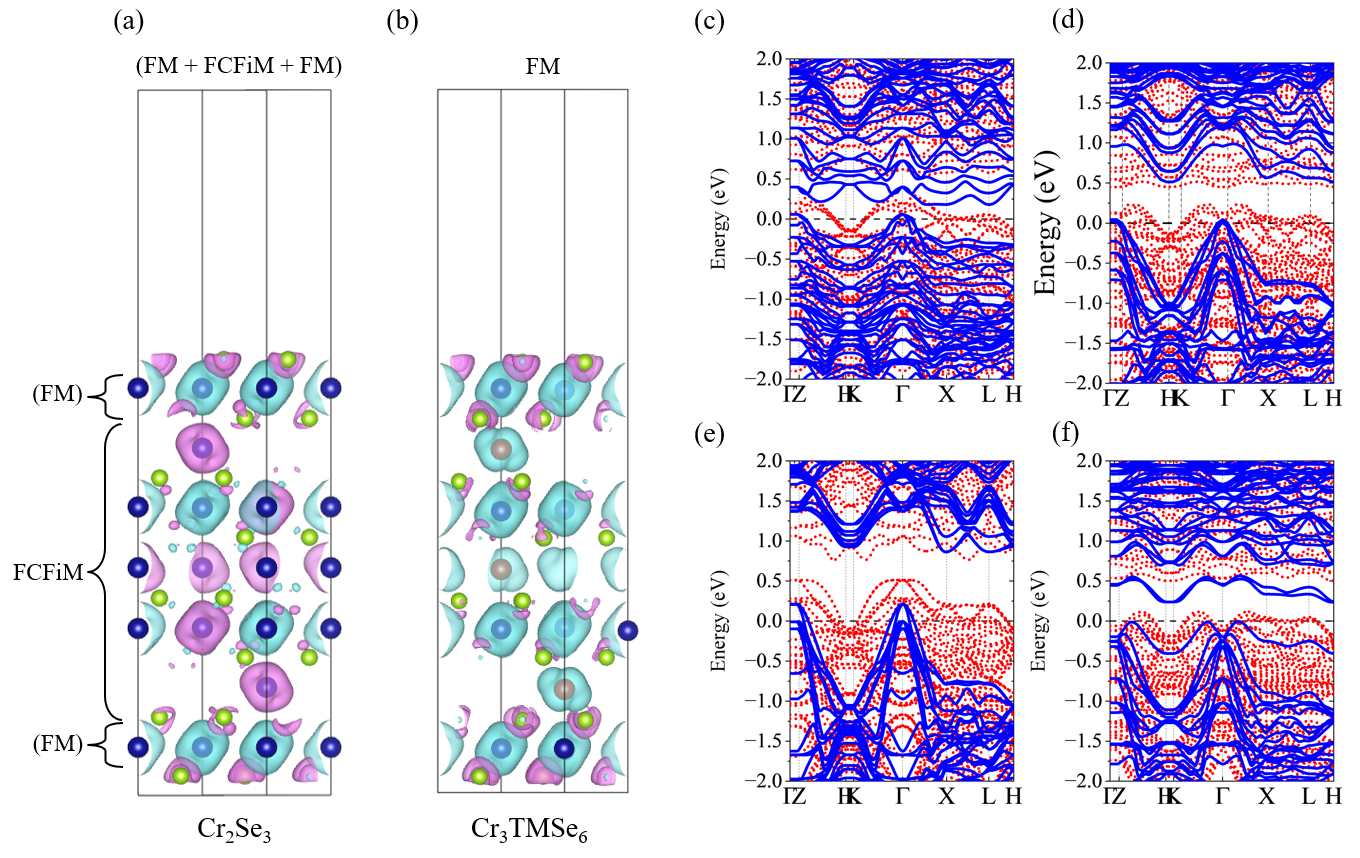


***Figure S5.*** *Spin-density distributions* ***(a and b)*** *and spin-polarized band structures* ***(c-f)*** *for monolayer Cr₃TMSe₆ (TM =* ***(c)****Cr,* ***(d)****V,* ***(e)****Mn, and* ***(f)****W. Cyan and Purple colors represent opposite spin signs. Blue solid and red dotted lines denote majority- and minority-spin channels, respectively, with the Fermi level set to zero energy. The monolayer limit induces notable changes in the electronic character relative to the bulk, as summarized in the main-text table. Note that the top and bottom layers of Cr_2_Se_3_ are ferromagnetically ordered upon scaling down the structure to a monolayer* ^3^. *Therefore, the fully compensated magnetic structure is encapsulated with FM top and bottom layers.*

# References

1 Aykol M, Dwaraknath SS, Sun W, Persson KA. Thermodynamic limit for synthesis of metastable inorganic materials. 2018.https://www.science.org.

2 Sun W, Dacek ST, Ong SP, Hautier G, Jain A, Richards WD *et al.* The thermodynamic scale of inorganic crystalline metastability. *Sci Adv* 2016; **2**. doi:10.1126/sciadv.1600225.

3 Shen J, Shen W, Lu Y, Wang F, Lin X, Lu Y. Spin-driven multiferroics in non–van der Waals Cr2S3. *Phys Rev B* 2025; **112**: 155416.
